# Ocean acidification and nitrate enrichment can mitigate negative effects of soft coral (*Xenia*) competition on hard coral (*Stylophora pistillata*) endosymbionts

**DOI:** 10.1038/s41598-025-15683-5

**Published:** 2025-08-15

**Authors:** Ana C. Grillo, Susana M. Simancas-Giraldo, Nico Steinel, Cybelle M. Longhini, Marcelo O. Soares, Sonia Bejarano, Guilherme O. Longo

**Affiliations:** 1 Leibniz Centre for Tropical Marine Research, 28359 Bremen, Germany; 2 Department of Oceanography and Limnology, Federal University of Rio Grande do Norte, Natal, 59014-002 RN Brazil; 3 College of Science and Engineering, James Cook University, Townsville, QLD 4811 Australia; 4 Helmholtz Centre for Polar and Marine Research, Alfred Wegener Institute, 27568 Bremerhaven, Germany; 5 Marine Ecology Department, University of Bremen, 28359 Bremen, Germany; 6 Institute of Marine Sciences, Federal University of Ceará, Fortaleza, 60165-081 CE Brazil

**Keywords:** Scleractinian corals, Malacalcyonacea, Eutrophication, Nutrient pollution, Climate change, Ecological interactions, Coral reefs, Community ecology

## Abstract

The combination of ocean acidification (OA) and eutrophication can undermine the physiological performance of reef-building corals during competition for benthic space, leading to shifts towards non-accreting organisms like soft corals. We conducted a 28-day laboratory orthogonal experiment to test if acidification (950 µatm *p*CO_2_) and moderate to high nitrate enrichment (4 and 8 µmolL^−1^) negatively affect the hard coral *Stylophora pistillata* while physically competing with the soft coral *Xenia* spp. We measured photosynthetic efficiency (PE) in hard corals and growth rate, Symbiodiniaceae density, and chlorophyll-*a* concentration in both hard and soft corals as proxies for their condition and responses to competition. Competition with the soft coral reduced PE, Symbiodiniaceae and chlorophyll-*a* contents of *S. pistillata*, while acidification alone and coupled with nitrate enrichment mitigated endosymbiont responses. The growth and chlorophyll-*a* concentrations of *Xenia* spp. were decreased by competition, but the soft coral was consistently benefited under nitrate enrichment. These results highlight that competition alone has a stronger negative impact on hard corals than on soft corals. Our study provides experimental evidence on how OA and eutrophication interact and shape coral dynamics, an overlooked but urgent topic in predicting reef futures under environmental change.

**Supplementary Information:**

The online version contains supplementary material available at 10.1038/s41598-025-15683-5.

## Introduction

Species interactions support entire ecosystems and are responsible for maintaining biodiversity and ensuring ecological services and functions^1^. Competition is one of the strongest interactions shaping communities both on land and in the sea, determining the configuration of species assemblages, and it is strongly affected by the environment^2,3^. Global climatic and local anthropogenic stressors can affect the responses to competition between species^3,4^, but these effects are not fully understood, and their interactions are rarely disentangled^5^. With increasing and worsening environmental changes affecting natural communities worldwide^6,7^, it is urgent to understand their unique and combined effects on competitive interactions. This could help to anticipate changes in ecosystem composition and functioning in response to future scenarios of increasing stressors, understand how newly dominant species can shape communities, and inform science-based management strategies to sustain the contributions of ecosystems to people.

Tropical coral reefs are among the most speciose and productive systems on Earth^8^. Space on the benthos is limited, and sessile reef organisms compete for it to settle and grow. The outcome of this array of competitive interactions ultimately determines benthic community structure^9^. Once dominant on coral reefs, reef-building corals are becoming progressively less abundant and are at risk of losing their ecological functions^7,10,11^, being in some cases replaced by non-reef builders (e.g., soft corals^12^). This has led to an increase in the frequency of competitive interactions between reef building corals and non-reef building organisms^13,14^. Understanding what mechanisms determine the results of this interaction is therefore crucial to anticipate the community structure of emerging reefs. This may ultimately determine whether the benthos will be structurally complex and accreting CaCO_3_ (e.g., if hard corals are abundant), or less topographically complex and non-accreting. Also, reefs dominated by non-reef builders can compromise the dispersal of wave energy and shoreline protection, turning coastlines more susceptible to erosion^15^. This information can then guide expectations on how these systems function and how to best preserve them.

Several mechanisms have evolved to facilitate the colonization of available space by sessile reef organisms and competition with neighboring taxa^13^. For instance, soft corals (Order Malacalcyonacea) display rapid asexual reproduction and growth rates that allow them to quickly monopolize free space and outcompete reef-building corals (Order Scleractinia) by overgrowing them^16–18^. Soft corals can also release allelopathic compounds that can harm the tissue of scleractinians and inhibit the settlement of their larvae^18,19^. In addition, depending on their morphology, competitors can shade photosynthetic organisms by overtopping^20,21^. Further, depending on their positioning relative to water flow and currents, branching soft corals can abrade the sensitive tissue of neighboring hard corals^21^.

In response to neighboring competitors, hard corals can defend their space using specialized tentacles, such as mesenterial filaments and sweeper tentacles, that can deter competitors by damaging and killing the tissue nearby^22^. Fast-growing hard corals can outcompete neighbors through overgrowth or overtopping^20^, and escape from competition by growing upwards^23^ or preventing their own growth in the margin of contact and redirecting it^24^ and using allelopathic compounds^13^. As the physiology of organisms is strongly dependent on abiotic variables, it is expected that the efficiency of competitive mechanisms in corals could be influenced by environmental stressors, such as increasing global temperature and ocean acidification^3^.

Coral reefs are currently exposed to a plethora of increasing anthropogenic stressors, such as more intense and frequent heatwaves, ocean acidification, deoxygenation, pollution, and overfishing^11^. While there is an extensive literature focusing on the separated and combined effects of ocean warming, acidification, and eutrophication on reef-building corals^25–27^, there is a lack of knowledge on how ocean acidification and nutrient pollution can affect competitive interactions sustained by corals^5^. While the ocean absorbs large amounts of atmospheric CO_2_^28^, seawater becomes more acidic, reducing the availability of carbonates and severely affecting biological calcification processes in reef builders^29,30^. Ocean acidification also reduces skeletal density and growth rates of hard corals^31^, making them more vulnerable to mechanical abrasion caused by contact with other organisms (e.g., soft corals^32^). Conversely, soft corals appear resistant to ocean acidification, given that their skeletal structures (i.e., calcite sclerites) are embedded in and protected by fleshy tissue^33,34^. Soft corals are reportedly abundant in naturally acidified locations^35^, which implies that this resistance could favor their dominance under future climate change scenarios.

Eutrophication, one of the most prevalent forms of anthropogenic pollution, includes higher inorganic nitrogen concentrations (e.g., nitrate) in seawater^36^ and has been linked to negative impacts on hard corals^37^. Elevated nitrate concentrations can reduce calcification and growth rates^36^, increase susceptibility to bleaching^38,39^, mortality^39^,and development of diseases^40,41^ in hard corals. However, depending on nutrient concentrations, it may also enhance the density of zooxanthellae and coral photosynthetic rates^42,43^. In soft corals, the effects tend to be less damaging^44^. The Xeniidae, for instance, are resistant to phosphate and organic enrichment^45,46^. In fact, community shifts leading to soft coral dominance have been associated with increases in nutrient pollution^12,47,48^. Ocean acidification and eutrophication may thus benefit opportunistic taxa (e.g., soft corals) that can outcompete hard corals for space^3,14^. Separate environmental stressors (e.g., acidification, warming, and nutrient pollution) have reportedly strengthened the competitive advantages of macroalgae and zoantharians over hard corals^49–51^, whereas other experimental studies report no effects of acidification on hard-soft coral competition and between hard corals^33^. Few experiments have investigated the effects of multiple and simultaneous stressors on corals and their competitive dynamics^52^. Specifically, the separate and combined effects of ocean acidification and eutrophication on the competition between hard and soft corals remain to be explored.

**Fig. 1:**
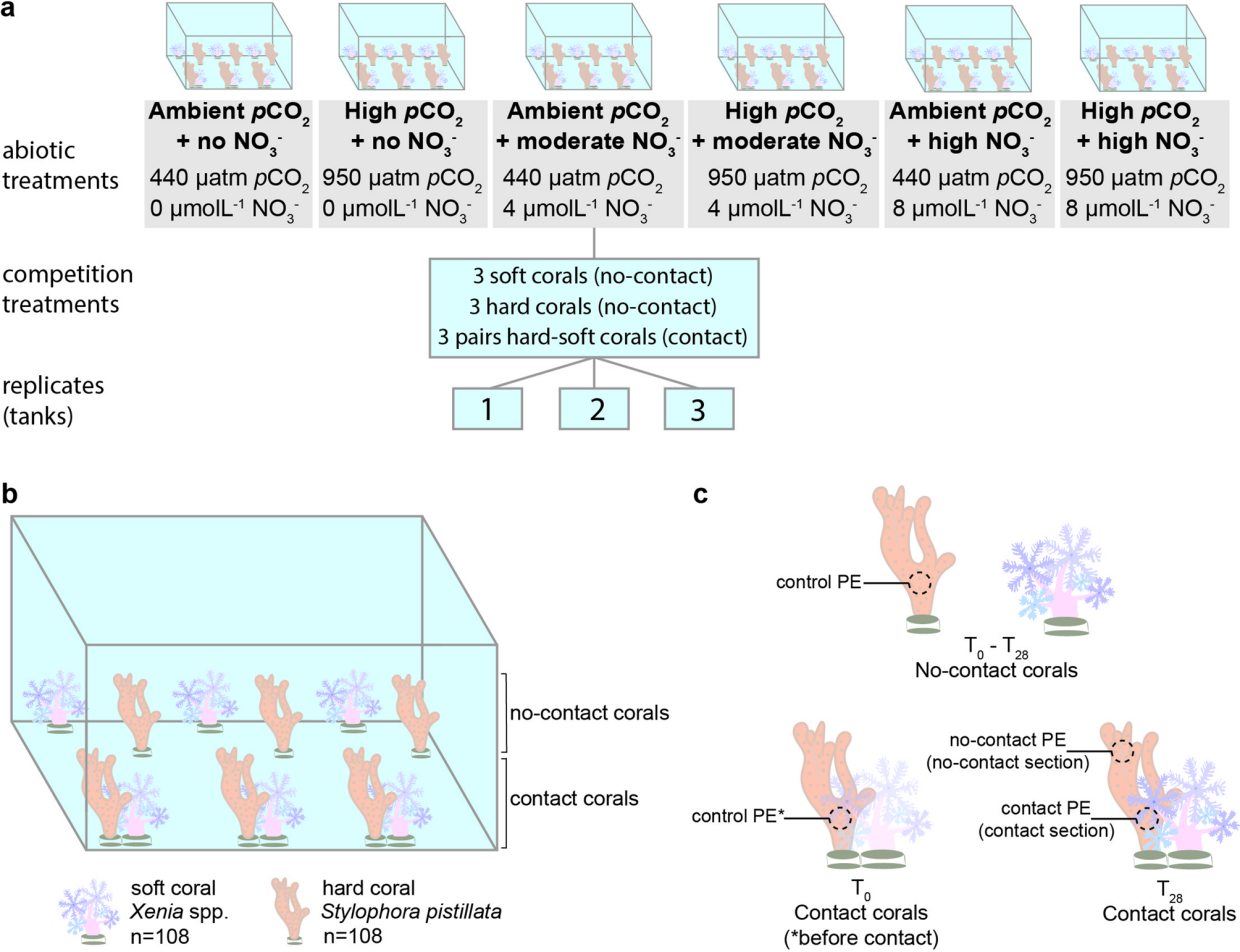
**Experimental design**.** a** Schematic representation of the experimental design. **b** Each tank contained 12 corals: three *Xenia* spp. colonies, three *Stylophora pistillata* colonies (no-contact corals) and three pairs of *Xenia* spp. physically contacting *S. pistillata* (contact corals; competition treatment). **c** Scheme representing photosynthetic efficiency (PE) readings on the hard coral *S. pistillata* on each competition treatment at the beginning (T_0_) and end (T_28_, after 28 days) of the experiment.

In this study, we assessed the effects of acidification and eutrophication on the competition between a hard and a soft coral. Specifically, we investigated the separate and combined effects of end-of-century ocean acidification levels (950 µatm *p*CO_2_^53^) and moderate to high nitrate enrichment (4 µmolL^−1^ − 8 µmolL^−1^ NO_3_−^26,54^) on the hard coral *Stylophora pistillata* and the soft coral *Xenia* spp. and on their competition for space. For this, we conducted an orthogonal mesocosm experiment with the hard coral *S. pistillata* physically contacting the soft coral *Xenia* spp. and exposed them to a total of six abiotic treatments for 28 days (Fig. 1). We measured selected physiological parameters of both corals that serve as proxies for assessing the treatment effects on their condition and on their responses to competition, namely the photosynthetic efficiency of *S. pistillata*, and growth rate, Symbiodiniaceae density, and chlorophyll-*a* concentration of both *S. pistillata* and *Xenia* spp.

## Results

### Seawater parameters

Mean *p*CO_2_ ranged from 470.55 ± 11.27 µatm in ambient *p*CO_2_ treatments to 962.29 ± 13.39 µatm in acidification treatments, whereas mean pH was at 7.78 ± 0.01 in acidification treatments and at 8.0 ± 0.01 in ambient *p*CO_2_ treatments (Table 1). These values differed between ambient and acidification treatments over time (LMM, post hoc, *p* < 0.01; Supplementary Figure S1). Carbonate concentration varied from 84.12 ± 2.49 µmolkg^−1^ in acidification treatments to 111.60 ± 4.39 µmolkg^−1^ in ambient *p*CO_2_ treatments, and aragonite saturation state varied from 1.34 ± 0.04 in acidification treatments to 1.78 ± 0.07 in ambient *p*CO_2_ treatments (Table 1, Supplementary Figure S1). Overall nitrate concentration in the controls for nitrate enrichment (0.20 ± 0.10 µmolL^−1^) was lower than concentrations reached under moderately enriched conditions (3.79 ± 0.14 µmolL^−1^) and in highly enriched tanks (8.18 ± 0.19 µmolL^−1^, Table 1, Supplementary Table S1). Values differed among nitrate enrichment treatments at the time of nitrate addition (LMM, post hoc, *p* < 0.01; Supplementary Figure S2), even though concentrations declined within 24 h after each addition.

**Table 1:** **Characterization of seawater parameters**. Measured and calculated seawater parameters (mean ± SE) for each acidification (*p*CO_2_) and nitrate enrichment (NO_3_^−^) treatment (abiotic treatments). Carbonate chemistry values are means of five measurements along the experiment and NO_3_^−^ values are means of four nutrient measurements over the experiment.

	Measured							Calculated				
Abiotic treatment	temperature °C	salinity psu	pH NBS	NO_3_^−^ µmolL^−1^	TA µmolkg^−1^	PO_4_ µmolkg^−1^	Si µmolkg^−1^	*p*CO_2_ µatm	CO_3_^2−^ µmolkg^−1^	HCO_3_^−^ µmolkg^−1^	CO_2_ µmolkg^−1^	Ω_Ar_
Ambient *p*CO_2_; no NO_3_^−^ (control)	25.61 ± 0.01	34.98 ± 0.04	7.951 ± 0.020	0.12 ± 0.08	1572.06 ± 50.76	0.13 ± 0.04	1.81 ± 0.59	501.43 ± 14.59	96.96 ± 6.61	1311.38 ± 35.79	14.02 ± 0.41	1.54 ± 0.11
High *p*CO_2_; no NO_3_^−^	25.59 ± 0.02	35.01 ± 0.04	7.798 ± 0.014	0.27 ± 0.19	1924.91 ± 61.02	0.62 ± 0.53	1.29 ± 0.33	920.05 ± 17.46	87.50 ± 4.92	1700.62 ± 50.45	25.71 ± 0.49	1.39 ± 0.08
Ambient *p*CO_2_; moderate NO_3_^−^	25.61 ± 0.01	34.98 ± 0.04	7.997 ± 0.023	3.86 ± 0.21	1678.58 ± 56.23	0.12 ± 0.03	0.80 ± 0.28	477.95 ± 24.04	113.49 ± 8.01	1378.40 ± 39.87	13.36 ± 0.67	1.81 ± 0.13
High *p*CO_2_; moderate NO_3_^−^	25.49 ± 0.01	34.97 ± 0.04	7.786 ± 0.016	3.72 ± 0.20	1961.27 ± 37.98	0.12 ± 0.03	0.59 ± 0.28	978.88 ± 25.18	86.51 ± 4.25	1740.86 ± 29.17	27.36 ± 0.71	1.38 ± 0.07
Ambient *p*CO_2_; high NO_3_^−^	25.53 ± 0.02	34.97 ± 0.04	8.042 ± 0.019	8.35 ± 0.30	1709.75 ± 39.20	0.14 ± 0.03	1.25 ± 0.51	432.28 ± 15.19	124.36 ± 6.85	1381.53 ± 27.00	12.08 ± 0.43	1.98 ± 0.11
High *p*CO_2_; high NO_3_^−^	25.53 ± 0.02	34.93 ± 0.04	7.763 ± 0.014	8.00 ± 0.25	1870.81 ± 40.09	0.13 ± 0.13	1.34 ± 0.57	987.95 ± 23.61	78.35 ± 3.55	1669.04 ± 33.07	27.62 ± 0.66	1.25 ± 0.06

TA: total alkalinity, *p*CO_2_: partial pressure CO_2_, CO_3_^2−^: carbonate concentration, HCO_3_^−^: bicarbonate concentration, Ω_Ar_ aragonite saturation state.

### Effects of acidification and nitrate enrichment on competition in *S. pistillata*

**Fig. 2:**
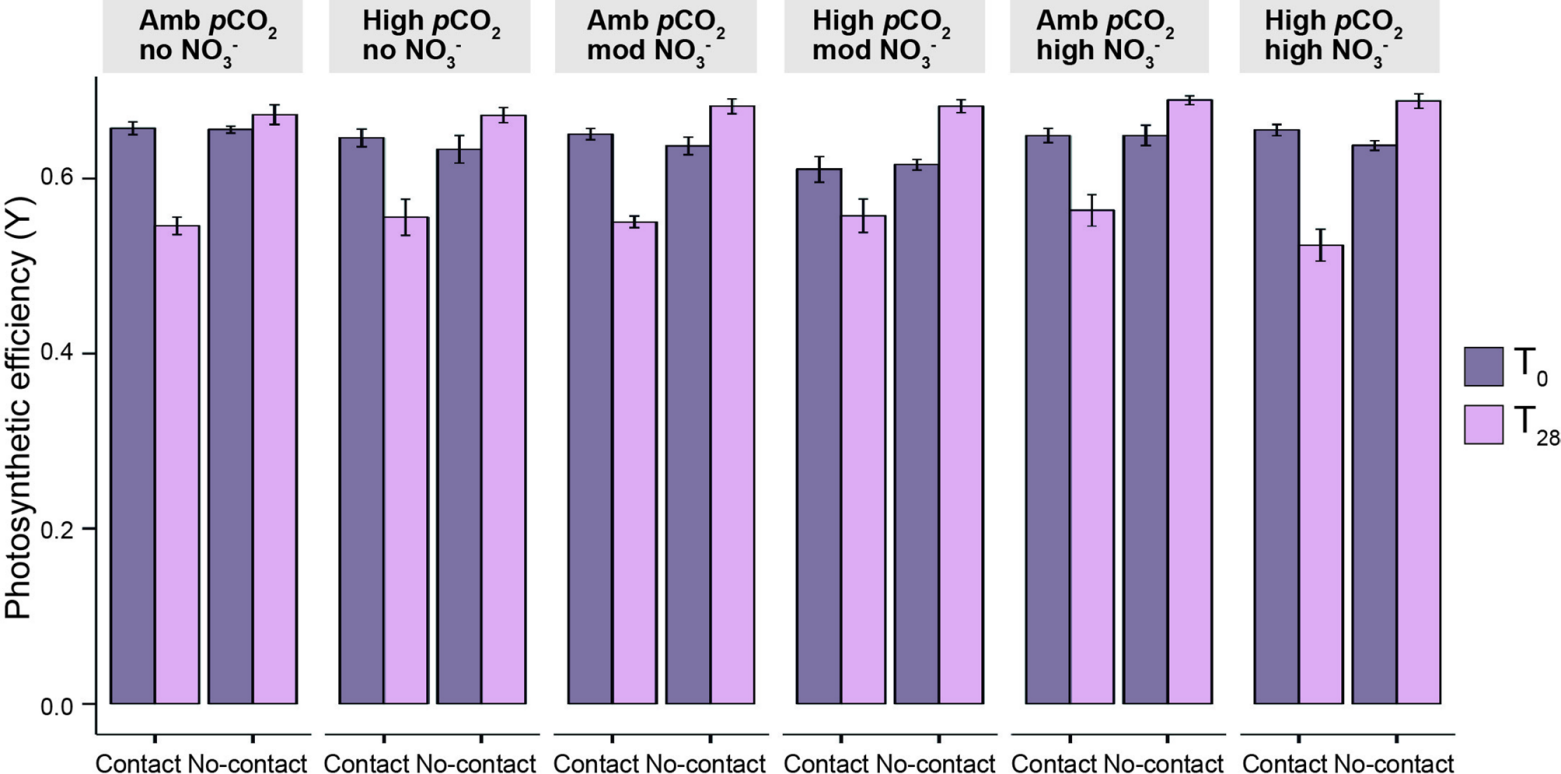
**Photosynthetic efficiency changes in *Stylophora pistillata * under competition, acidification, and nitrate enrichment.** Photosynthetic efficiency (effective quantum yield, Y, mean ± SE) of the hard coral *Stylophora pistillata* competing (contact) and not competing (no-contact; competition treatments) with the soft corals within each abiotic treatment (acidification and nitrate enrichment), at the beginning (T_0_) and end (T_28_) of the experiment. Amb = ambient, mod = moderate treatment concentrations.

**Fig. 3:**
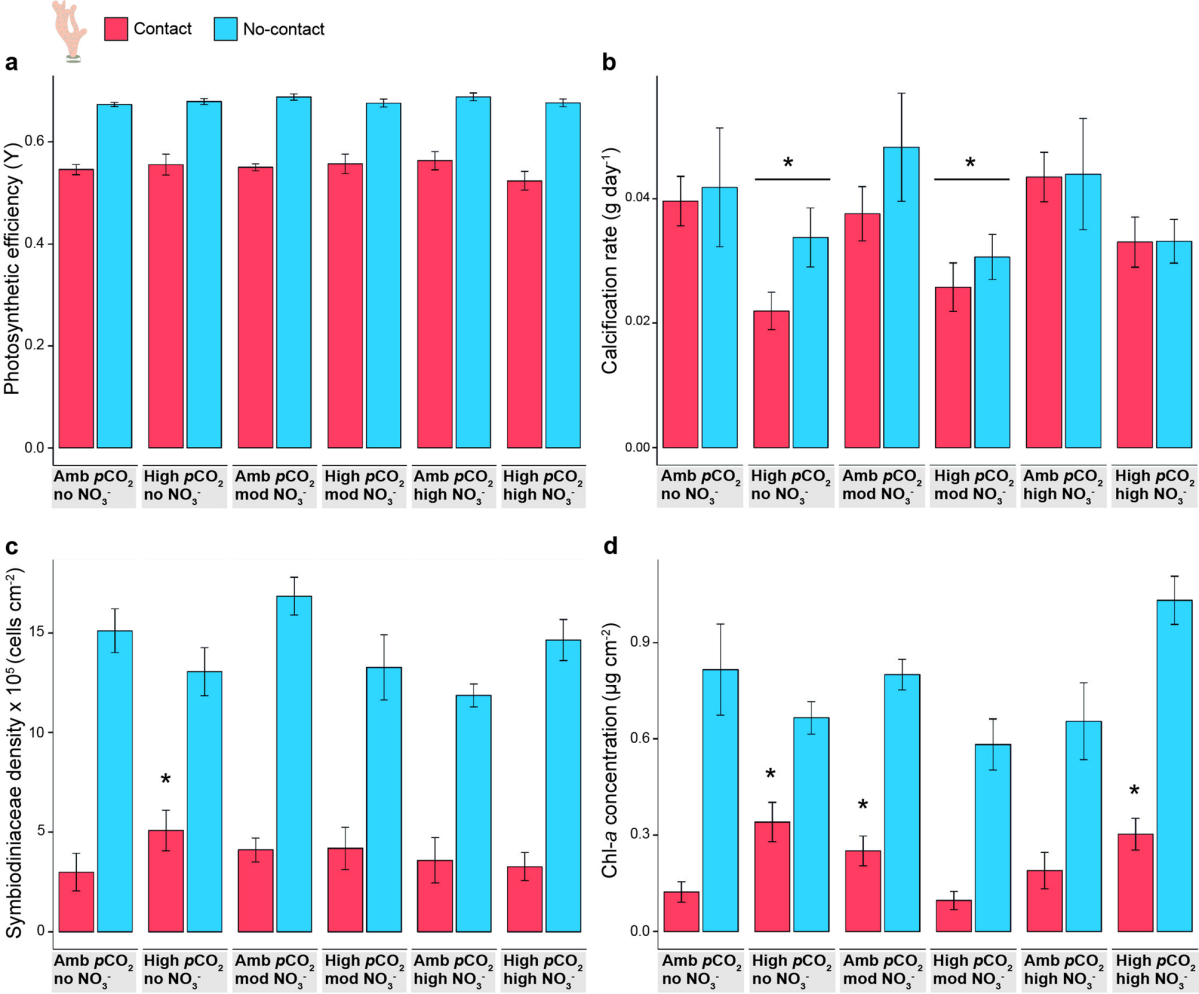
**Physiological responses of *Stylophora pistillata*** **to competition, acidification, and nitrate** **enrichment.** Physiological measurements of the hard coral *Stylophora pistillata* competing (contact) and not competing (no-contact; competition treatments) with the soft corals within each abiotic treatment (acidification and nitrate enrichment). **a** Photosynthetic efficiency (effective quantum yield, Y, mean ± SE) of contact and no-contact sections of competing hard corals at T_28_; **b** calcification rate (g day^−1^, mean ± SE) of contact and no-contact hard corals; **c** Symbiodiniaceae density (cells cm^−2^, mean ± SE) of contact and no-contact sections of competing hard corals; **d** chlorophyll-*a* concentration (µg cm^−2^, mean ± SE) of contact and no-contact sections of competing hard corals. Asterisks indicate significant differences compared to the control treatment (GLMM, *p* < 0.05); Amb = ambient, mod = moderate treatment concentrations.

The effects of treatments on hard corals were quantified by measuring four key physiological parameters that serve as proxies for their condition: photosynthetic efficiency, calcification rate, Symbiodiniaceae density, and chlorophyll-*a* concentration. Neither acidification nor nitrate enrichment affected the responses to competition for *S. pistillata* in terms of photosynthetic efficiency (PE; Figs. 2 and 3a). Regardless of *p*CO_2_ level or nitrate concentration, competition consistently reduced the mean PE of hard corals contacting soft corals by 15% from the beginning (0.645 ± 0.004) to the end (0.549 ± 0.007) of the experiment. PE of *S. pistillata* corals not contacting soft corals was 19% higher than PE of hard corals contacted by soft corals at the end of the experiment (0.681 ± 0.003, GLMM, *p* < 0.001; Fig. 2). No significant interactive effect of acidification, nitrate enrichment, and competition on PE was detected (GLMM, *p* > 0.05, Supplementary Table S2a).

At the end of the experiment, neither acidification nor nitrate enrichment affected the PE of hard corals regardless of whether these were in contact with soft corals or not (Supplementary Fig. S3, GLMM, *p* > 0.05) and of different sections of hard corals contacted by soft corals (Fig. 3a, GLMM, *p* > 0.05, Supplementary Table S2b). The sections of *S. pistillata* that remained in contact with *Xenia* spp. suffered a reduction in PE in T_28_ (Fig. 3a) throughout all abiotic treatments. The magnitude of this reduction was similar across acidification or nitrate enrichment treatments (GLMM, *p* > 0.05, Supplementary Table S2c). Mean PE within the sections of *S. pistillata* that were in contact with *Xenia* spp. was 19% lower than within no-contact sections (0.549 ± 0.007 and 0.680 ± 0.003, respectively), and these differences remained consistent across treatments (GLMM, *p* < 0.001, Tukey, *p* < 0.001, Supplementary Table S3a). The PE of hard corals not in contact with soft corals was unaffected by acidification or nitrate enrichment throughout the experiment (0.638 ± 0.004 in T_0_ and 0.681 ± 0.003 in T_28_).

Competition with soft corals did not affect the calcification rate of the hard coral *S. pistillata* within any of the abiotic treatments (GLMM, *p* > 0.05, Supplementary Table S2d). Acidification alone and coupled with moderate nitrate concentrations significantly reduced the calcification rate of *S. pistillata* irrespective of competition (Fig. 3b, GLMM, *p* = 0.004 and *p* = 0.034, respectively, Supplementary Table S2d). The mean calcification rate of all hard corals exposed to those treatments was reduced by 30% compared to the control treatment (0.028 ± 0.003 g day^−1^ for both acidification treatments, 0.041 ± 0.005 g day^−1^ for the control treatment). In the absence of nitrate enrichment, acidification halved the calcification rate of competing *S. pistillata* compared to competing hard corals exposed to high nitrate enrichment coupled with ambient *p*CO_2_ levels (Tukey, *p* = 0.037, Supplementary Table S3b; 0.022 ± 0.003 and 0.044 ± 0.004 g day^−1^, respectively).

At the end of the experiment, competition reduced Symbiodiniaceae densities in hard corals across all treatments (Fig. 3c, GLMM, *p* < 0.001, Supplementary Table S2e). Sections in *S. pistillata* that were in contact with soft corals had 70% lower mean Symbiodiniaceae densities than sections that were not in contact (3.873 ± 0.372 and 14.136 ± 0.501 × 10^5^ cells cm^−2^, respectively). These differences were consistent regardless of acidification or nitrate enrichment treatments (Tukey, *p* < 0.001, Supplementary Table S3c). Acidification alone enhanced Symbiodiniaceae densities in competing hard corals compared to hard corals exposed to ambient *p*CO_2_ levels (GLMM, *p* = 0.032, Supplementary Table S2e). In acidified conditions, the mean Symbiodiniaceae densities within hard coral tissue placed in contact with soft corals were 1.7-fold higher than what the same part of the corals under ambient *p*CO_2_ levels (5.078 ± 1.017 and 2.994 ± 0.946 × 10^5^ cells cm^−2^, respectively).

Competing with *Xenia* spp. during 28 days decreased mean chlorophyll-*a* concentrations in *S. pistillata* across treatments (Fig. 3d, GLMM, *p* < 0.001, Supplementary Table S2f). Sections in hard corals in contact with soft corals had 70.5% less mean chlorophyll-*a* concentrations than sections not in contact (0.224 ± 0.023 and 0.760 ± 0.041 µg cm^−2^, respectively, Supplementary Table S3d). Acidification increased chlorophyll-*a* concentrations in *S. pistillata* competing with *Xenia* spp. compared to ambient *p*CO_2_ levels (GLMM, *p* < 0.05, Supplementary Table S2f). Mean chlorophyll-*a* concentrations of contact hard corals under acidification alone and coupled with high nitrate concentrations were 2.6-fold higher than under the control treatment (0.341 ± 0.061, 0.303 ± 0.049, and 0.124 ± 0.032 µg cm^−2^, respectively). Moderate nitrate concentrations coupled with ambient *p*CO_2_ levels also doubled the chlorophyll-*a* concentrations in competing *S. pistillata* compared to the control treatment (0.251 ± 0.047 and 0.124 ± 0.032 µg cm^−2^, respectively).

### Effects of ocean acidification and nitrate enrichment on competition in *Xenia* spp.

**Fig. 4:**
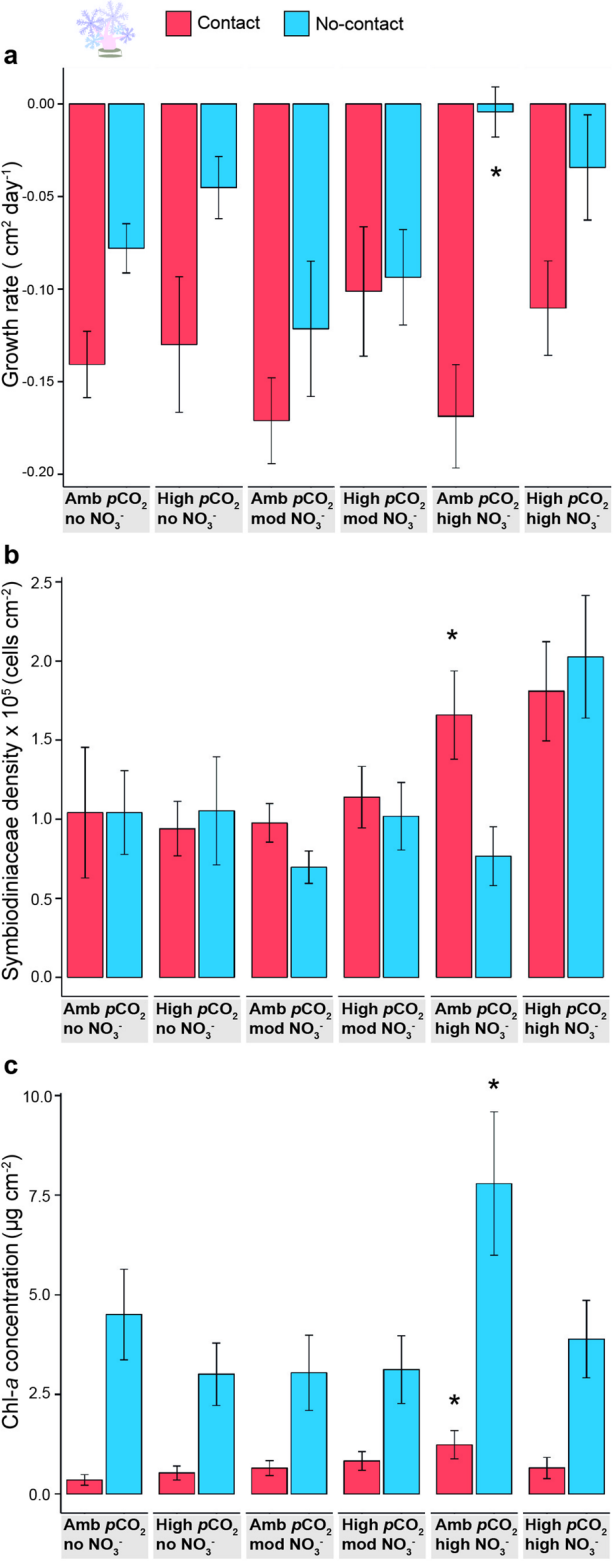
**Physiological responses of * Xenia*** **spp. to competition, acidification, and nitrate** **enrichment**. Physiological measurements of the soft coral *Xenia* spp. competing (contact) and not competing (no-contact; competition treatments) with the hard corals, for each abiotic treatment (acidification and nitrate enrichment). **a** Mean growth rate (cm^2^ day^−1^ ± SE); **b** Symbiodiniaceae density (cells cm^−2^, mean ± SE); **c** chlorophyll-*a* concentration (µg cm^−2^, mean ± SE). Asterisks indicate significant differences compared to the control treatment (GLMM, *p* < 0.05); Amb = ambient, mod = moderate treatment concentrations.

For the soft corals, we quantified the effects of treatments by measuring their growth rate, Symbiodiniaceae density, and chlorophyll-*a* concentration. Throughout the experiment, the growth rate of most *Xenia* spp. soft coral colonies decreased regardless of whether they were positioned in direct contact or no contact with hard corals and irrespective of experimental treatment, pointing to a general negative response to the experimental tanks (Fig. 4a). Significant differences in growth rates between competing and non-competing soft corals across abiotic treatments were found (GLMM and Tukey, *p* < 0.05, Supplementary Table S4a, S5a). The mean growth rate of no-contact *Xenia* spp. underwent a decrease of 0.063 ± 0.011 cm^2^ day^−1^, while contact *Xenia* spp. reduced by 0.137 ± 0.012 cm^2^ day^−1^. Under high nitrate concentrations, the decrease in the growth rate of *Xenia* spp. that were not competing with hard corals was less pronounced than for soft corals exposed to the control treatment (Fig. 4a, GLMM, *p* = 0.011, Supplementary Table S4a). The mean growth rate of no-contact *Xenia* spp. exposed to no nitrate enrichment (control treatment) was 18-fold lower than no-contact colonies under high nitrate concentrations coupled with ambient *p*CO_2_ (−0.078 ± 0.013 and − 0.004 ± 0.014 cm^2^ day^−1^, respectively).

Competition did not have a negative effect on the Symbiodiniaceae density of *Xenia* spp. across treatments (Fig. 4b, Supplementary Table S4b). High nitrate enrichment increased Symbiodiniaceae densities in soft corals when contacting hard corals compared to non-competing soft corals in the control treatment (GLMM, *p* = 0.015, Supplementary Table S4b). Under high nitrate concentrations coupled with ambient *p*CO_2_ levels, mean Symbiodiniaceae densities in competing *Xenia* spp. increased by 37% compared to non-competing *Xenia* spp. in the control treatment (1.659 ± 0.280 and 1.042 ± 0.264 × 10^5^ cells cm^−2^, respectively). Chlorophyll-*a* concentrations differed between competing and non-competing *Xenia* spp. across abiotic treatments (GLMM, *p* < 0.05, Supplementary Table S4c, S5b). The mean chlorophyll-*a* concentration in competing *Xenia* spp. was 80% lower than in non-competing *Xenia* spp. (0.708 ± 0.1 and 4.225 ± 0.497 µg cm^−2^, respectively). Nitrate enrichment increased chlorophyll-*a* concentrations in *Xenia* spp. compared to control conditions (Fig. 4c, GLMM, *p* < 0.05). Under high nitrate concentrations coupled with ambient *p*CO_2_ levels, contact and no-contact *Xenia* spp. had an increase of 70 and 40%, respectively, in mean chlorophyll-*a* concentrations compared to soft corals exposed to no nitrate enrichment (1.234 ± 0.354 and 0.352 ± 0.134 µg cm^−2^ for contact, 7.792 ± 1.801 and 4.503 ± 1.136 µg cm^−2^ for no-contact, in nitrate enrichment and control treatments, respectively). None of the *Xenia* spp. colonies underwent bleaching or tissue necrosis throughout the experiment.

## Discussion

Anticipating how once scleractinian-dominated reefs are reconfiguring and functioning under anthropogenic pressures requires a mechanistic understanding of the effects of different factors in coral responses to competition. This study provides experimental evidence for the separate and combined effects of a major climate-driven pressure (i.e., ocean acidification) and a prevalent local-scale stressor (i.e., nitrate enrichment) on physiological responses to competition between the hard coral *S. pistillata* and the soft coral *Xenia* spp. Competition harmed *S. pistillata* colonies only in the contacted section of their tissue, and the abiotic treatments either had no effect or mitigated these negative impacts, depending on their combination, concentrations, and the physiological parameters considered. The PE, Symbiodiniaceae densities, and chlorophyll-*a* concentrations of hard corals were significantly reduced by competition regardless of abiotic treatments. Calcification rates of hard corals were negatively affected by acidification treatments regardless of being competing or not with soft corals. PE of hard corals was reduced only when competing with soft corals, and neither *p*CO_2_ levels nor nitrate concentrations influenced this pattern. Acidification, both alone and in combination with moderate nitrate enrichment, reduced the calcification rates in both competing and non-competing hard corals, whereas acidification alone appeared to mitigate the effect of competition on hard coral Symbiodiniaceae densities and chlorophyll-*a* concentrations. The latter were also favored by acidification coupled with high nitrate enrichment. While competition decreased the growth rates and chlorophyll-*a* concentrations of *Xenia* spp. across abiotic treatments, all physiological parameters of the soft corals were favored under high nitrate enrichment alone.

Overall, *S. pistillata* was negatively affected when competing with *Xenia* spp., and the damage was restricted to the contact sections of the hard corals. For instance, the PE, Symbiodiniaceae densities, and chlorophyll-*a* concentrations of competing *S. pistillata* were lower within the sections in contact with the soft coral than in adjacent sections. Both physical and chemical competition are known to damage hard corals in direct contact with soft corals, causing tissue discoloration and/or bleaching, declines in PE and Symbiodiniaceae densities, and tissue necrosis^13,18^. *Stylophora pistillata* is, in fact, ranked as an inferior competitor relative to other coral species^55^. Competition with *S. pistillata* did not cause negative effects on Symbiodiniaceae densities of *Xenia* spp., despite reducing their chlorophyll-*a* concentrations. Soft corals, including *Xenia* spp., grow rapidly, reproduce asexually, have high fecundity, and can release allelopathic compounds, all of which makes them strong benthic competitors^16,17^. These traits enable soft corals to quickly preempt reef space, limiting the establishment of hard corals by inhibiting larvae settlement^19^ or overgrowing coral colonies^18^, thus leading to community shifts^48,56^.

Coral responses to competition can vary under changing environmental conditions^49–51^, highlighting the importance of investigating competitive interactions under different stressors. Here, we demonstrate that responses to competition between *S. pistillata* and *Xenia* spp. could either be affected or not by ocean acidification and/or nitrate enrichment, depending on the abiotic and physiological parameters considered. Acidification coupled with no or moderate nitrate enrichment suppressed the calcification rate of both competing and non-competing *S. pistillata*. Ocean acidification affects hard coral growth rates, by decreasing aragonite saturation state and reducing the availability of carbonate in the water^29,30^. The responses of *S. pistillata* in our experiment agree with the expected influence of acidification and were confirmed in high *p*CO_2_ treatments, presenting the lowest water carbonate concentrations and aragonite saturation states. Competition can reportedly inhibit skeletal growth in hard corals^57^. In an intraspecific competition experiment with scleractinian corals subjected to acidification, competition inhibited the growth of corals, and this was exacerbated under acidified conditions^58^, whereas other experiments have found that competition decreased calcification rates in hard corals more than acidification^59,60^. In our experiment, we did not find clear evidence that competition reduced the calcification rate of hard corals. Instead, acidification negatively affected the calcification rate of all *S. pistillata*, irrespective of competition.

Here, high nitrate enrichment under ambient *p*CO_2_ levels enhanced the calcification rate of competing *S. pistillata* when compared to an acidification scenario in the absence of nitrate. Although some studies have found deleterious effects of elevated nitrate concentrations on hard corals, like increased bleaching^39^ and reduced calcification^42^, there is evidence of a positive growth response^26,61^. It possible that the nitrate input in our experiment was rapidly taken up either by microorganisms in the water or within the coral holobiont, which could have affected the limited differences in the calcification rates between hard corals with high and no nitrate enrichment under ambient *p*CO_2_ levels that we found. Conversely, ambient *p*CO_2_ coupled with moderate and high nitrate concentrations led to the highest levels of carbonate concentrations and aragonite saturation states among all treatments (i.e., prerequisites for coral calcification), suggesting that our high nitrate enrichment treatment favored the calcification of competing hard corals when compared to corals under acidification, helping them withstand competition. This is consistent with previous studies indicating that nitrate input can benefit corals when subjected to stressful environmental conditions (i.e., elevated temperature and *p*CO_2_^26,62^). These responses could also be explained by the enhancement of primary production by symbionts, maintaining lower CO_2_ and higher pH conditions at a cellular level, subsequently favoring the carbonate precipitation by hard corals^63^. However, it is important to note that we did not manipulate phosphate in our experiment, which might have led to phosphate deprivation in the hard corals, potentially leading to negative impacts on the symbionts and in physiological functions that we did not detect or measure^64,65^.

The growth rate of all *Xenia* spp. colonies was unaffected by acidification alone or when combined with different nitrate concentrations. This contrasts with another study which found a growth rate reduction in *X. umbellata* colonies exposed to acidification, probably because a different growth metric was used (number of polyps^66^). Our results agree with the findings of a previous experiment showing that acidification did not impact the growth of soft corals^33^. Different from scleractinians, some octocorals deposit calcite sclerites within their tissue, which could protect these skeleton structures from external conditions^34^. This most likely explains the unaltered physiological parameters of soft corals when subjected to acidification.

Despite their ecological importance and high abundance in Indo-Pacific and Red Sea reefs, soft corals of the Xeniidae family remain taxonomically challenging, with more than 20 recorded species and some still undescribed^67^. This taxonomic uncertainty complicates ecological interpretations, as competitive superiority and invasiveness potential could vary among species^67^. Although our experiments included two *Xenia* species, potentially introducing a confounding factor in the treatment effects, it is unlikely that this was the primary driver of the differences we observed, as including *Xenia* species in our statistical model did not change the results. Moreover, the observed decrease in chlorophyll-*a* concentration of competing *Xenia* spp. compared to non-competing *Xenia* spp. across all abiotic treatments suggests that, in this case, species-specific differences do not override the overall trends driven by competition or environmental conditions.

While nutrient input was expected to increase the growth of soft corals and enhance their competitive response, our results do not support this expectation. Field observations have shown positive effects of eutrophication, including dissolved inorganic nitrogen and suspended particulate matter, on the dominanceof soft corals in reefs^47,68^ which could also lead to community phase shifts^12^. However, as in our experiment we only manipulated nitrate, phosphatecould have been a limiting nutrient for the physiological processes of corals, like primary production, as both nutrients are needed for their growth^69^. Another caveat of our experiment was that the growth rate of most soft corals, irrespective of competition, was reduced over time and even in the control treatment. This signals an unknown tank effect that could not be controlled and affected *Xenia* spp. colonies. In addition to that, competing *Xenia* spp. shrank more than those that were not competing. Nevertheless, under high nitrate enrichment alone, *Xenia* spp. was least affected compared to other treatments, pointing to the positive effect of nutrient enrichment on this organism. Under a possible tank effect that was unfavorable for the development of *Xenia* spp., nitrate enrichment could have alleviated the negative tank consequences and benefited the growth of soft corals. In sum, acidification had a negative effect on the growth rate of *S. pistillata* and a neutral effect on *Xenia* spp., for both competing and non-competing corals. While high nitrate enrichment benefitted the growth of competing hard corals, we are unable to affirm this for the competing soft corals. However, as some Xeniids can grow and reproduce faster than hard corals in nature^17^, high nitrate inputs could benefit their abundance and indirectly impact hard corals by increasing the frequency of competition between these organisms^47^.

While competing hard corals exhibited a local decline in Symbiodiniaceae densities and chlorophyll-*a* concentrations across all treatments, high *p*CO_2_ levels improved these numbers in *S. pistillata* when competing with *Xenia* spp. Few studies have shown trends for increasing Symbiodiniaceae densities^70^ and chlorophyll-*a* concentrations^71^ under acidification conditions, and most have found no influence^72^. In our experiment, acidification coupled with high nitrate enrichment and moderate nitrate enrichment alone also stimulated chlorophyll-*a* concentrations in competing *S. pistillata*. There is evidence of dissolved inorganic nitrogen input increasing responses of symbionts, as they use this nutrient for their growth^61^. However, no other studies have addressed how these responses can be affected by the interacting effects of acidification, nitrate enrichment, and competition. We were unable to find differences in the PE of competing *S. pistillata* as they were all reduced in similar magnitudes across treatments. In acidified scenarios, net production in corals could be enhanced because Symbiodiniaceae are released from carbon limitation, using it as a source for photosynthesis^73^. However, in our study, the overall PE reduction due to competition might have uncovered the effects of abiotic treatments on competing hard corals. Even with reduced PE, Symbiodiniaceae, and chlorophyll-*a* amounts, our results evidence that acidification stimulated these responses, and corals subjected to high *p*CO_2_ levels could have achieved higher productivity under competition.

Competition did not have a negative effect on Symbiodiniaceae densities in soft corals. Interestingly, high nitrate enrichment resulted in higher Symbiodiniaceae densities in competing *Xenia* spp. compared to non-competing *Xenia* spp. exposed to the control treatment. This points to a positive effect of high nitrate enrichment on the competitive responses of *Xenia* spp. Conversely, competition reduced chlorophyll-*a* concentrations in soft corals across all treatments, but high nitrate enrichment enhanced their concentrations in both competing and non-competing soft corals. An increase in symbiont population densities and chlorophyll-*a* contents under eutrophication is in accordance with other studies as algal symbionts are released from nitrogen limitation^42^. The incongruence between high Symbiodiniaceae densities and low chlorophyll-*a* concentrations was previously reported for hard corals^50^ and octocorals^74^. Evidence suggests that symbionts can photoacclimate as a response to changing conditions and chlorophyll contents react quickly to these changes^73,75^, helping explain the negative effects of competition solely on chlorophyll-*a* concentrations in our study. We did not measure photosynthetic activity in our soft corals, but as we did not record loss of algal symbionts or tissue bleaching, this suggests that *Xenia* spp., irrespective of competition or species, were functional. Overall, acidification improved the density of Symbiodiniaceae and chlorophyll-*a* levels in competing *S. pistillata*, and chlorophyll-*a* concentrations were also improved by the combination of acidification and high nitrate enrichment, and moderate nitrate enrichment alone. For *Xenia* spp., although we cannot confirm if high nitrate enrichment favored its competitive responses in terms of chlorophyll-*a* concentrations, our results point to this treatment enhancing their Symbiodiniaceae densities when competing.

A limitation of our study is that we used few genotypes of aquarium-acclimated hard and soft corals. We acknowledge that this is not ideal, but the fact that the fragments were blindly interspersed among treatments may have addressed the low genetic variability issue. If there were any genotype effects related to a non-random distribution pattern, we would expect to see a tank effect in the analysis or consistent responses rather than variable responses to each treatment. Although including more genotypes would certainly help to better understand population-level variability, it is unlikely that it would change the overall direction of the physiological responses observed. Also, we recognize that long-term aquarium acclimation may influence coral baseline physiology compared to wild colonies, but it also ensured healthy, standardized conditions, and we expect the direction of competitive responses under eutrophication and acidification to remain consistent due to fundamental physiological mechanisms.

This experiment demonstrates the combined effects of ocean acidification and nitrate enrichment influencing the physiological responses of hard and soft corals under competition. Our results are particularly important in a scenario with increasing global and local anthropogenic stressors, where studies that focus on simultaneous abiotic variables at different levels can help disentangle their impacts on the dynamics of coral reefs. Understanding how these variables can affect specific physiological traits of competing corals is essential due to the current degradation of coral reefs and overall decrease in abundance of hard corals and increase of dominant fast-growing competitors^7,10,11,48^. In our study, the cost of competition for the hard coral *S. pistillata* under control and manipulated ocean acidification and nitrate enrichment treatments was higher than for the soft coral *Xenia* spp. Specific effects on both corals depended on the combination of the abiotic variables, their levels, and the physiological parameters considered. Under future scenarios of environmental change that tend to undermine hard corals more than their soft-bodied competitors, ocean acidification and nitrate enrichment may play an important role in determining whether reefs will be dominated by reef-builders.

## Methods

### Study species sourcing and maintenance

The study focused on the reef-building coral *S. pistillata* (Esper 1797) (Hexacorallia: Scleractinia: Pocilloporidae) and the soft coral *Xenia* spp. (Octocorallia: Malacalcyonacea: Xeniidae) as these are widely distributed in a variety of reef habitats of the Indo-Pacific and Red Sea^16^. *Stylophora pistillata* is a branching species often used as a model in ecological studies, while *Xenia* spp. are frequently found covering extensive areas on the reefs and competing with hard corals^17,56^.

A laboratory experiment was conducted between November 2022 and January 2023 at the Marine Experimental Ecology Laboratory (MAREE) of the Leibniz Centre for Tropical Marine Research (ZMT) in Bremen, Germany. Two aquarium-reared colonies of *S. pistillata* were sourced through the Hagenbeck Zoo, Germany, in 2018, and since then several fragments were cut and reared in aquaria at the ZMT. For this experiment, we propagated fragments of these colonies (*n* = 108) in sizes of ~ 5 cm height and glued them onto ceramic plugs with bases ~ 2 cm diameter (Fauna Marin, Germany) with Coral Glue (EcoTech Marine, USA). Posteriorly, these fragments were blindly interspersed among treatments. Colonies of *Xenia* spp. (*n* = 108), ~ 4 cm long and aquarium-reared, were purchased from DeJong Marinelife Coral Nursery (Netherlands) already attached to ceramic discs with bases ~ 4.7 cm in diameter. Molecular identification through PCR sequencing of the mitochondrial gene mtMutS (i.e., an equivalent to the COI gene in other animal taxa^76^) confirmed that the *Xenia* spp. obtained corresponded to an unidentified species from the Indo-pacific region reported as *Xenia* sp. 4^67^ and *Xenia umbellata* (Lamarck 1816), a common xeniid endemic of the Red Sea region. Soft corals will hereinafter be referred to as *Xenia* spp. All fragments of hard and soft corals were allowed to recover for two months inside two separate 500-L recirculating tanks prior to the experiment. These tanks were maintained at 25.5 °C, 35 psu, and 330 µmol photons m^−2^ s^−1^ for temperature, salinity, and light levels respectively, and we used artificial seawater in the entire experiment (Pro-Reef Sea Salt, Tropic Marin, Germany).

## Experimental design

Following recovery, the base of the corals’ plugs and discs was brushed to remove any fouling algae or invertebrates and then transferred to 40-L experimental glass tanks (*n* = 18 tanks). Each experimental tank contained a thermostat heater (EHEIM thermocontrol 75 watts, Germany) to ensure temperature remained stable at 25.5 °C, a water pump (TUNZE Turbelle nanostream 6025 and 6045, Germany) covered with a mesh for flow reduction, a grid to fit the corals’ plugs and discs, a 5-mm diameter plastic tube connected to a microporous ceramic air stone for CO_2_ infusion, a 5-mm diameter soft plastic tube for water sampling, a 4-mm plexiglass lid to reduce water evaporation and loss of CO_2_, and LED lamps (Aquaillumination, Hydra 52 HD, USA, in a 12 h:12 h light/dark cycle reaching a maximum irradiance of 330 µmol photons m^−2^ s^−1^).

A total of 12 corals mounted on their plugs and discs were randomly positioned inside each tank (six hard corals and six soft corals avoiding physical contact among them) and left to acclimatize for ten days in the following abiotic conditions: temperature 25.56 ± 0.01 °C, salinity 34.73 ± 0.02 psu, pH 8.21 ± 0.01, dissolved oxygen 7.1 ± 0.13 mgL^−1^ (mean ± SE).

### Effects of ocean acidification and nitrate enrichment in hard-soft coral competition

Following acclimation, three pairs of corals within each tank (three hard corals and three soft corals) were positioned in physical contact with one another to simulate spatial competition (competing corals), and six other corals (three hard corals and three soft corals) remained separate from one another (non-competing corals). Corals were subjected to six different abiotic treatments resulting from the combination of two ocean acidification (*p*CO_2_) levels and three nitrate enrichment (NO_3_^−^) concentrations, with three replicate tanks per treatment (*n* = 18 tanks, Fig. 1): (i) ambient *p*CO_2_ and no nitrate enrichment (“control treatment”, 440 µatm *p*CO_2_, 0 µmolL^−1^ NO_3_^−^); (ii) high *p*CO_2_ and no nitrate enrichment (950 µatm *p*CO_2_, 0 µmolL^−1^ NO_3_^−^); (iii) ambient *p*CO_2_ and moderate nitrate concentration (440 µatm *p*CO_2_, 4 µmolL^−1^ NO_3_^−^); (iv) high *p*CO_2_ and moderate nitrate concentration (950 µatm *p*CO_2_, 4 µmolL^−1^ NO_3_^−^); (v) ambient *p*CO_2_ and high nitrate concentration (440 µatm *p*CO_2_, 8 µmolL^−1^ NO_3_^−^); (vi) high *p*CO_2_ and high nitrate concentration (950 µatm *p*CO_2_, 8 µmolL^−1^ NO_3_^−^).

The three nitrate concentrations simulated an oligotrophic environment (no or very low NO_3_^−^, < 2 µmolL^−1^, here named as “no nitrate enrichment”^77)^, a moderate (~ 4 µmolL^−126^), and a high nitrate enrichment (~ 8 µmolL^−1 54^). Nitrate enrichment was obtained from a sodium nitrate solution (1.36 gL^−1^ NaNO_3_, 13 and 26 mL of solution for moderate and high nitrate concentration treatments, respectively) pipetted directly in the experimental tanks three times a week, reaching the desired levels. This simulated episodic pulses of enrichment since the concentrations of nitrate decreased with time. Two scenarios were simulated for the ocean acidification treatments: a present-day greenhouse gas emission (ambient ~ 8 pH and *p*CO_2_ ~ 440 µatm, here named as “ambient *p*CO_2_ level”) and a high greenhouse gas emission scenario projected for the end-of-century under the Intergovernmental Panel on Climate Change scenario^53^ (SSP3-7.0, ~ 7.8 pH and *p*CO_2_ ~ 900–1000 µatm, here named as “high *p*CO_2_ level” or “acidification treatment”). The gas mixing system was composed of a compressor (AGRE, Germany) that generated an airflow that first passed through a limestone column (AquaCare, Germany) to produce depleted CO_2_ air. Then, the air was enriched with CO_2_ to the desired levels in the GasMix system (HTK, Germany; two concentration channels were used). The airflow was regulated by needle valves before bubbling into the experimental tanks through plastic tubes with ceramic airstones. Experimental laboratory conditions were maintained and monitored for 28 days. This duration has been shown to be sufficient to observe the effects of competition, acidification, and nutrient enrichment in corals^26,52^.

During the acclimation and experimental phases, seawater salinity was adjusted whenever necessary by adding reverse-osmosis water (Two Stage Reverse Osmosis Unit High Pressure 2-150, Aquacare, Germany); 10% of the water of each tank was exchanged three times a week with new artificial seawater, and tanks were cleaned every ten days with a 75% water exchange. Corals were fed twice a week during the acclimation and the experimental phases with harvested *Artemia* sp. and were observed capturing it during the feeding sessions (~ 7 mL of life food culture of *Artemia* sp. per tank, ~ 60 individuals per 10 µL). Water exchanges and cleaning were always done before nutrient addition, and corals were fed on different days from nutrient addition to avoid interfering with nitrate concentrations. Physical parameters were measured every one to three days using a multiparameter probe (WTW Multi 3430 m, Germany) connected to sensors for conductivity and temperature (TetraCon 925), dissolved oxygen (FDO925), and pH (SenTix 980, Supplementary Table S1). Light irradiance was measured using a light meter data logger (LI-250 A, LI-COR, USA, with a quantum sensor MQS-B, Walz, Germany).

### Monitoring water nutrient and carbonate chemistry

Water samples for nitrate quantification were collected four times during the experiment, from each experimental tank, 20 min after adding the nutrient and was measured spectrophotometrically (Infinite M200 NanoQuant Plate Tecan, Switzerland) using a colorimetric technique^78^ (Supplementary Table S1). Samples were filtered through CA filters (0.45 μm), collected into 60 mL syringes, and then transferred into 15 mL centrifuge tubes. An additional four samples were collected 24 h after nutrient addition to check for declines in nitrate concentrations over time.

To characterize the seawater carbonate chemistry, water samples were collected five times throughout the experiment between 7h00 and 09h00 from within each experimental tank (Supplementary Table S1). Temperature, salinity, and pH (NBS scale) were measured in each experimental tank minutes after water collection using the multiparameter probe (WTW). Total alkalinity (TA) was determined spectrophotometrically (Infinite M200 NanoQuant Plate, Tecan, Switzerland) with a color dye (bromophenol blue) and formic acid^79^ using certified standards for alkalinity (Hach, USA). The dissolved inorganic nutrients phosphate (PO_4_) and silicate (Si) were also analyzed by colorimetry and quantified spectrophotometrically^78^. Seawater carbonate chemistry parameters (i.e., *p*CO_2_, carbonate concentration [CO_3_^2−^], bicarbonate concentration [HCO_3_^−^], aqueous CO_2_, and aragonite saturation state Ω_Ar_) were calculated using a model of ionic associations using TA, pH, temperature, salinity, and nutrients (PO_4_ and Si) as input data. The CO2SYS3.0^80^ was employed using Lueker values for constants^81^.

### Measuring physiological responses of *S. pistillata*

Photosynthetic efficiency (PE; effective quantum yield) of the Symbiodiniaceae within hard corals was measured through their chlorophyll *a* fluorescence (ΔF/Fm′) at the beginning (T_0_) and end (T_28_) of the experiment using a Diving Pulse-Amplitude Modulated (PAM) Fluorometer (Walz, Germany). PE values range from 0 to 1, with numbers around or higher than 0.6 indicating a high photosynthetic efficiency and lower values pointing to the loss of algal symbionts or photosynthetic pigments, which could compromise the health of corals^82^. Measurements were taken under light conditions and with the same lighting levels at both times to maintain a constant light absorbance by corals. At T_0_, for all contact and no-contact hard corals, we took one reading at the center of each coral (control PE). At T_28_, for the no-contact corals, we took one reading on the center of the coral, representing the final PE value. For the contact corals, we took two readings per sample: one on a control section that was not in contact with the soft coral, usually near the upper extremity (no-contact PE value, no-contact section), and another on the section that was in contact with the soft coral (contact PE value, contact section; Fig. 1c). All measurements were performed ensuring a constant distance between the probe and the coral colony being measured.

The growth rate of hard corals was assessed through their calcification rate using the buoyant weight technique^83^. Coral samples were weighed in seawater at T_0_ and T_28_ of the experiment, together with the ceramic plugs as they were already glued, using an analytical balance (Sartorius ME235S, Germany). Seawater density was accurately determined by measuring salinity and temperature (kept stable at 25 °C using a heating circulator). To obtain the skeletal density, at the end of the experiment, six coral samples per treatment (i.e., two per experimental tank, non-competing corals) were soaked in a sodium hypochlorite solution until all their tissue was removed, then rinsed and left in seawater. Afterward, they were buoyantly weighed as described above, and then oven-dried overnight at 50 °C to assess their dry weight and the mean density^84^. The mean value for each treatment was used to calculate the final dry weight of coral samples^84^. The calcification rate per day during the experiment was obtained as the difference in dry weight between the final and initial times divided by 28 days (g day^−1^).

At the end of the experiment, hard corals were fragmented to obtain Symbiodiniaceae densities and chlorophyll-*a* concentrations. Two fragments were obtained from the front side of each colony of contact corals (total *n* = 108): one removed from the section that was contacting the soft coral, and another from an adjacent section that was not contacting the soft coral (considered the control for the competition treatment). These fragments were flat and bidimensional, allowing us to photograph them with a scale to estimate their surface area, while minimizing potential errors associated with the analysis using the software ImageJ^85^. Then, the fragments were incubated for 1 h in 1 M NaOH on an orbital shaker and vortexed until the entire tissue was separated from the skeleton. The remaining skeleton was removed, and the solution was centrifuged (3000 RCF, 5 min, 25 °C). The supernatant was discarded, and 1 mL of 1 M phosphate buffered saline solution (PBS) was added to the samples to dissolve the pellets, which were centrifuged again, and the supernatant discarded. Another 1 mL of 1 M PBS was added, and the samples were vortexed to homogenize the final pellets before two subsamples (0.5 mL) were taken for chlorophyll-*a* and Symbiodiniaceae counts. A drop of Lugol was added to the Symbiodiniaceae samples that were stored at 4 °C until the hemocytometer count (Improved Neubauer chamber, depth 0.1 mm) through microscopy, and data was normalized to the surface area of the coral fragments, estimated through photographs as described above. The chlorophyll-*a* samples were once more centrifuged as previously detailed, the supernatant was discarded, and the final pellets were frozen at −80 °C. Later, under minimal light exposure, the pellet was extracted by adding 1,5mL 96% ethanol, the solution was vortexed, heated for 30 min at 78 °C, and centrifuged (14000 RPM, 30 min, 4 °C). The supernatant samples were kept on ice until the absorbances were spectrophotometrically measured (665 and 750 nm, Spark Tecan plate reader, Switzerland), with three replicate readings per sample. Chlorophyll-*a* concentrations were calculated^86^ and normalized to the surface area of coral fragments.

### Measuring physiological responses of *Xenia* spp.

The growth rate of *Xenia* spp. throughout the experiment was estimated through changes in their surface area. All soft corals (*n* = 108) were photographed from the top with a size scale at T_0_ and T_28_, after being carefully left to rest in a tray filled with seawater for 3–5 min to minimize interferences in their measurements, and their areas were measured using ImageJ^85^. The growth rate of soft corals was calculated as the difference between the final and initial surface areas divided by the 28 days of the experiment to obtain an extension or shrinkage in area (cm^2^) per day.

All *Xenia* spp. samples (*n* = 108) were frozen at −80 °C upon finishing the experiment to evaluate the density of Symbiodiniaceae and chlorophyll-*a* concentrations. For each analysis, a subsample between ~ 1.5 and 3 cm^2^ was taken from each colony, ensuring that this was a representative portion after other studies conducted with soft corals^87,88^.The subsamples were photographed with a size reference to estimate their surface area using ImageJ^85^; afterwards they were homogenized mechanically using Eppendorf micro pestles, and the resulting tissue slurry volume was recorded and diluted in 1 mL of Milli-Q water. The diluted samples were centrifuged twice (1400 RPM, 10 min), and each time the obtained pellets were reserved. Symbiodiniaceae density was subsequently quantified under the microscope, resuspending the pellets in 1 mL Milli-Q water and placing a 10 µL aliquot in a cell counting chamber (Improved Neubauer chamber, depth 0.1 mm). To quantify the chlorophyll-*a* concentration within the coral tissue, 2 mL of 90% acetone were added to the pellets reserved for this analysis and allowed to stand overnight at 4 °C. Sample manipulations and chlorophyll-*a* extractions were conducted in the dark^88,89^. The extraction products were measured spectrophotometrically (630, 647, 664, and 750 nm, UV-1800 Shimadzu, Japan), the chlorophyll pigments were calculated^90^, and the results obtained for each quantification were normalized to the surface area of the corals.

## Statistical analyses

All statistical tests were performed using R in RStudio (R Core Team v. 4.2.3). To assess whether carbonate chemistry variables (pH, total alkalinity, *p*CO_2_, CO_3_^2−^, HCO_3_^−^, and CO_2_; response variables) differed between ambient and acidification treatments over time (fixed predictors), we fitted separate Linear Mixed-Effects Models (LMMs) using *lme4* package (v.1.1.36) accounting for each experimental tank as random predictor, followed by pairwise comparisons with the *emmeans* package (v.1.11.0). We assessed differences in nitrate concentrations (response variable) among the three nitrate treatments (no, moderate, and high enrichment) over time (fixed factors) using the same analysis.

To test whether there is an effect of competition on hard and soft corals and whether the hard vs. soft coral competition is influenced by acidification or nitrate enrichment or their combination (abiotic treatments), different Generalized Linear Mixed Models (GLMMs) were fitted according to each physiological parameter measured on hard and soft corals, as detailed below (i.e., PE, growth rate, Symbiodiniaceae density, chlorophyll-*a* concentration as response variables). For every model, the abiotic and competition treatments were considered as fixed predictors, and each experimental tank was considered as a random predictor. Upon finding significant differences, pairwise contrasts were performed using Tukey tests.

To test for the effect of abiotic and competition treatments on the PE values of hard corals (response variable), whilst accounting for the repeated measures made at two time points within the experiment (T_0_ and T_28_), we fitted a GLMM with a Beta distribution (required when data takes values between 0 and 1). To test for the effect of acidification and nitrate enrichment on competition for hard corals, we first compared the PE values of hard corals (response variables) in contact with soft corals with those positioned separately (i.e., no-contact) and among the different abiotic treatments in T_28_. For hard corals in contact with soft corals, we also compared the PE in T_28_ within sections in contact and in no-contact with soft corals and among abiotic treatments. For both analyses, we fitted separate GLMMs with Beta distribution. Additionally, for hard corals in contact with soft corals, we calculated the difference in PE between the contact and no-contact sections in T_28_ to test if the PE was equally affected among abiotic treatments using a GLMM with Gaussian distribution (fitting for data with normal distribution). To test the effects of abiotic and competition treatments on the calcification rate of hard corals (response variable), we fitted a GLMM with a Gamma distribution (suitable for positive continuous values). To test for the effects on Symbiodiniaceae density and chlorophyll-*a* concentration (response variables), we fitted separate GLMMs with Log-normal distributions (as an alternative to Gamma distribution) after removing three and eight data points identified as outliers, respectively.

We tested whether soft coral growth rate, Symbiodiniaceae densities, and chlorophyll-*a* concentrations (response variables) were affected by the experimental treatments fitting separate GLMMs with Gaussian, Gamma, and Log-normal (zero-inflated) distributions, respectively, after three outliers were removed from the Symbiodiniaceae dataset. For all three tests, we included the experimental tanks and *Xenia* species as random predictors. Tests were run using *glmmTMB* package v. 1.1.8 for GLMMs and *multicomp* package v.1.4.25 for Tukey tests. Model diagnostics were carried out using *DHARMa* package v.0.4.7 employing graphical assessments of the models’ residuals, quantile deviations, dispersion, and outliers. Outliers were identified and removed based on residual diagnostics using the same package, improving model assumptions. Statistical significance was considered when *p*-values were lower than 0.05.

## Supplementary Information

Below is the link to the electronic supplementary material.

Supplementary Material 1

## Data Availability

All data used in this manuscript is available in the JCU Research Data repository https://doi.org/10.25903/yjhm-db37 .
