# Supplementary material for: Ocean acidification and nitrate enrichment can mitigate negative effects of soft coral (Xenia) competition on hard coral (Stylophora pistillata) endosymbionts
Source: Sci Rep. 2025 Aug 15;15:29937. doi: 10.1038/s41598-025-15683-5 (PMC12356902; doi:10.1038/s41598-025-15683-5)
Supplement: Supplementary file 1 — Supplementary Material 1 [file 41598_2025_15683_MOESM1_ESM.pdf]

Supplementary Information

Ocean acidification and nitrate enrichment can mitigate negative effects of soft coral (*Xenia*) competition on hard coral (*Stylophora pistillata*) endosymbionts

Ana C. Grillo, Susana M. Simancas-Giraldo, Nico Steinel, Cybelle M. Longhini, Marcelo O. Soares, Sonia Bejarano, Guilherme O. Longo

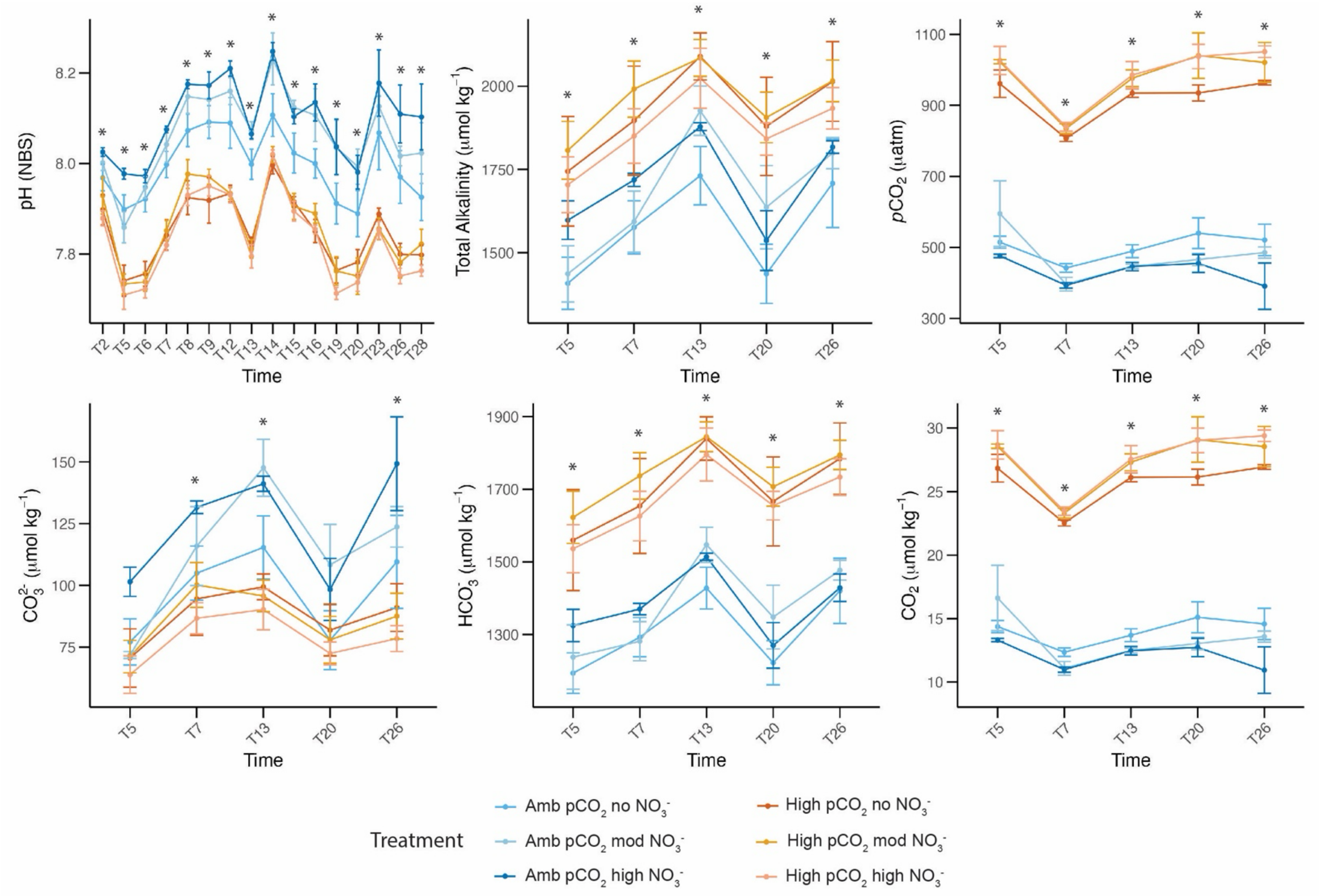

Supplementary Figure S1. Carbonate chemistry parameters. Mean carbonate chemistry parameters (± SE) for each abiotic treatment over time. Asterisks indicate significant differences between ambient and acidification treatments for each measurement time (LMM, post hoc, $p < 0.05$).

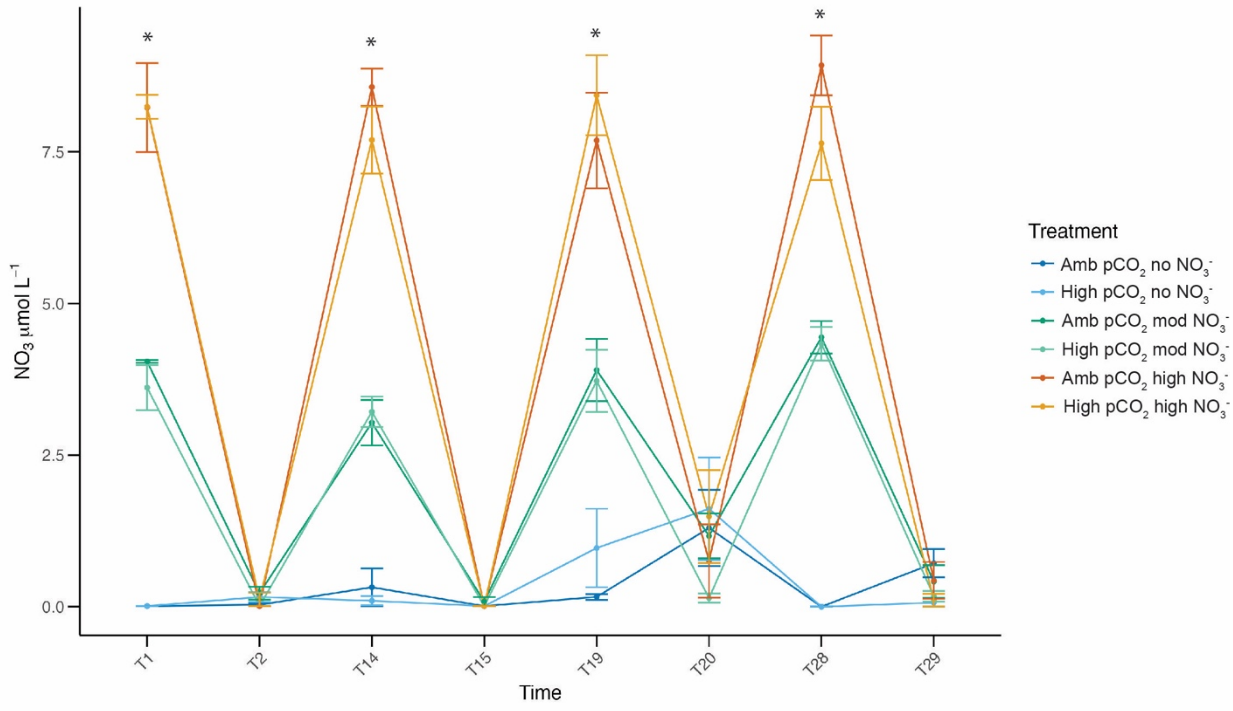

Supplementary Figure S2. Nitrate enrichment concentrations. Mean nitrate concentrations (± SE) for each abiotic treatment over time. Asterisks indicate significant differences among nitrate enrichment treatments for each measurement time (LMM, post hoc, $p < 0.05$). Note that nitrate concentrations were measured eight times during the experiment (at the time of nitrate addition and 24h later, as shown in the graph), but nitrate was added three times per week to the experimental tanks during the experiment (i.e., 13 times in total).

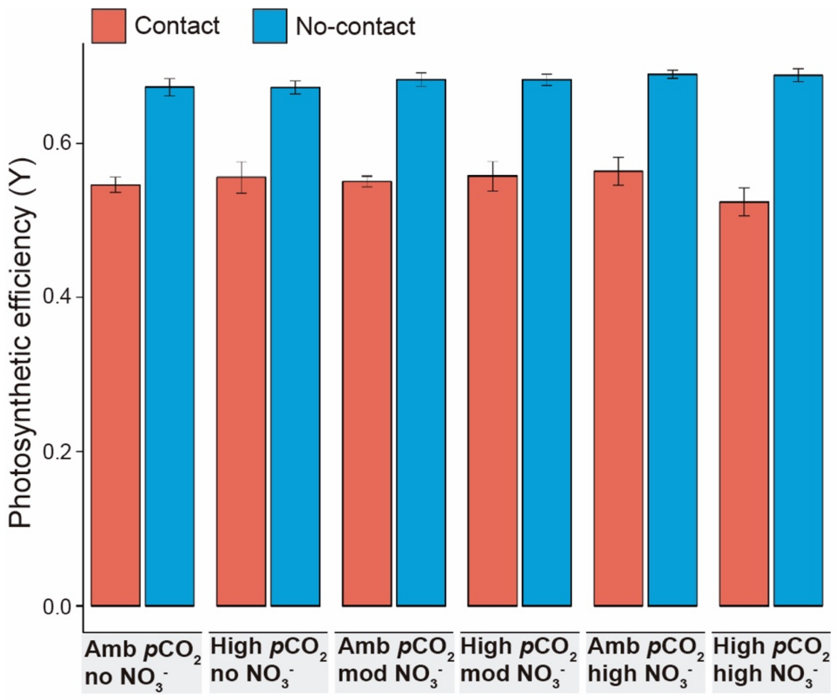

Supplementary Figure S3. Photosynthetic efficiency of *Stylophora pistillata*. Mean photosynthetic efficiency (effective quantum yield, Y, ± SE) at the end of the experiment for competing (contact) and non-competing (no-contact) hard corals (competition treatments) for each abiotic treatment (acidification and nitrate enrichment); Amb = ambient, mod = moderate treatment concentrations.

Supplementary Table S1. Seawater parameters monitoring. Seawater parameters measured along the experiment for each abiotic treatment.

Time	Abiotic treatment	Salinity psu	Temp °C	pH NBS	Dissolved oxygen mg L^{-1}	TA µmolkg^{-1}	PO_4 µmolkg^{-1}	Si µmolkg^{-1}	NO_3^- µmolL^{-1}
T_1	Amb pCO_2 no NO_3^-	-	-	-	-	-	-	-	0.01
	High pCO_2 no NO_3	-	-	-	-	-	-	-	0.01
	High pCO_2 High NO_3	-	-	-	-	-	-	-	8.24
	Amb pCO_2 High NO_3	-	-	-	-	-	-	-	8.23
	High pCO_2 Mod NO_3	-	-	-	-	-	-	-	3.61
	Amb pCO_2 Mod NO_3	-	-	-	-	-	-	-	4.04
T_2	Amb pCO_2 no NO_3^-	35.03	25.60	7.97	6.90	-	-	-	-
	High pCO_2 no NO_3	35.03	25.60	7.90	6.98	-	-	-	-
	High pCO_2 High NO_3	34.87	25.53	7.88	6.97	-	-	-	-
	Amb pCO_2 High NO_3	34.97	25.50	8.03	6.91	-	-	-	-
	High pCO_2 Mod NO_3	34.93	25.50	7.93	7.02	-	-	-	-
	Amb pCO_2 Mod NO_3	35.20	25.57	8.00	6.92	-	-	-	-
T_5	Amb pCO_2 no NO_3^-	35.13	25.57	7.90	6.79	1408.04	0.0	2.57	-
	High pCO_2 no NO_3	35.13	25.57	7.74	6.86	1744.10	0.0	1.91	-
	High pCO_2 High NO_3	35.03	25.50	7.71	6.87	1704.20	0.1	2.19	-
	Amb pCO_2 High NO_3	35.07	25.57	7.98	6.84	1597.45	0.0	3.71	-
	High pCO_2 Mod NO_3	35.10	25.50	7.73	6.78	1807.25	0.0	0.01	-
	Amb pCO_2 Mod NO_3	35.13	25.57	7.86	6.87	1436.98	0.0	0.76	-
T_6	Amb pCO_2 no NO_3^-	35.13	25.60	7.92	6.76	-	-	-	-
	High pCO_2 no NO_3	35.17	25.60	7.76	6.81	-	-	-	-
	High pCO_2 High NO_3	35.03	25.53	7.72	6.78	-	-	-	-
	Amb pCO_2 High NO_3	35.07	25.50	7.97	6.74	-	-	-	-
	High pCO_2 Mod NO_3	35.10	25.50	7.74	6.70	-	-	-	-
	Amb pCO_2 Mod NO_3	35.13	25.57	7.95	6.81	-	-	-	-
T_7	Amb pCO_2 no NO_3^-	35.17	25.63	8.00	6.89	1575.87	0.12	2.70	-

	High pCO_2 no NO_3	35.17	25.60	7.84	6.93	1896.63	0.12	1.80	-
	High pCO_2 High NO_3	35.07	25.57	7.82	6.98	1850.24	0.19	3.34	-
	Amb pCO_2 High NO_3	35.17	25.57	8.08	6.99	1718.36	0.20	1.15	-
	High pCO_2 Mod NO_3	35.20	25.50	7.85	7.07	1991.58	0.15	2.52	-
	Amb pCO_2 Mod NO_3	35.13	25.63	8.04	7.00	1593.47	0.16	2.45	-
T_8	Amb pCO_2 no NO_3^-	35.30	25.63	8.07	6.87	-	-	-	-
	High pCO_2 no NO_3	35.30	25.60	7.92	6.92	-	-	-	-
	High pCO_2 High NO_3	35.17	25.57	7.93	7.02	-	-	-	-
	Amb pCO_2 High NO_3	35.23	25.50	8.18	7.07	-	-	-	-
	High pCO_2 Mod NO_3	35.23	25.50	7.98	7.24	-	-	-	-
	Amb pCO_2 Mod NO_3	35.30	25.57	8.15	6.98	-	-	-	-
T_9	Amb pCO_2 no NO_3^-	35.20	25.63	8.09	7.01	-	-	-	-
	High pCO_2 no NO_3	35.20	25.63	7.92	6.92	-	-	-	-
	High pCO_2 High NO_3	35.10	25.60	7.95	6.98	-	-	-	-
	Amb pCO_2 High NO_3	35.17	25.57	8.17	7.06	-	-	-	-
	High pCO_2 Mod NO_3	35.10	25.53	7.97	7.46	-	-	-	-
	Amb pCO_2 Mod NO_3	35.20	25.63	8.14	6.93	-	-	-	-
T_{12}	Amb pCO_2 no NO_3^-	35.03	25.70	8.09	7.07	-	-	-	-
	High pCO_2 no NO_3	35.07	25.60	7.93	6.97	-	-	-	-
	High pCO_2 High NO_3	34.87	25.60	7.93	7.19	-	-	-	-
	Amb pCO_2 High NO_3	34.97	25.57	8.21	7.11	-	-	-	-
	High pCO_2 Mod NO_3	34.90	25.57	7.93	7.20	-	-	-	-
	Amb pCO_2 Mod NO_3	35.00	25.60	8.16	6.98	-	-	-	-
T_{13}	Amb pCO_2 no NO_3^-	34.87	25.60	8.00	6.87	1731.11	0.20	1.13	-
	High pCO_2 no NO_3	34.97	25.57	7.83	6.93	2089.54	0.13	1.25	-
	High pCO_2 High NO_3	34.90	25.50	7.80	6.93	2023.58	0.22	0.57	-
	Amb pCO_2 High NO_3	34.97	25.50	8.07	6.90	1879.02	0.25	0.44	-
	High pCO_2 Mod NO_3	34.83	25.47	7.81	6.88	2085.19	0.21	0.34	-

	Amb pCO_2 Mod NO_3	34.93	25.60	8.07	6.92	1926.25	0.15	0.28	-
T_{14}	Amb pCO_2 no NO_3^-	35.03	25.63	8.11	7.04	-	-	-	0.32
	High pCO_2 no NO_3	35.10	25.63	8.00	7.01	-	-	-	0.10
	High pCO_2 High NO_3	35.00	25.53	8.02	7.14	-	-	-	7.70
	Amb pCO_2 High NO_3	35.10	25.53	8.25	7.17	-	-	-	8.57
	High pCO_2 Mod NO_3	34.93	25.50	8.02	7.16	-	-	-	3.21
	Amb pCO_2 Mod NO_3	35.07	25.63	8.23	7.20	-	-	-	3.03
T_{15}	Amb pCO_2 no NO_3^-	34.97	25.63	8.02	6.88	-	-	-	-
	High pCO_2 no NO_3	34.97	25.60	7.92	6.90	-	-	-	-
	High pCO_2 High NO_3	34.83	25.53	7.89	6.93	-	-	-	-
	Amb pCO_2 High NO_3	34.87	25.53	8.10	6.93	-	-	-	-
	High pCO_2 Mod NO_3	34.87	25.53	7.91	6.95	-	-	-	-
	Amb pCO_2 Mod NO_3	34.97	25.60	8.12	6.90	-	-	-	-
T_{16}	Amb pCO_2 no NO_3^-	35.07	25.63	8.00	6.95	-	-	-	-
	High pCO_2 no NO_3	35.07	25.63	7.85	6.92	-	-	-	-
	High pCO_2 High NO_3	34.93	25.57	7.85	7.06	-	-	-	-
	Amb pCO_2 High NO_3	34.97	25.53	8.14	7.05	-	-	-	-
	High pCO_2 Mod NO_3	35.00	25.53	7.89	7.14	-	-	-	-
	Amb pCO_2 Mod NO_3	35.07	25.63	8.11	7.02	-	-	-	-
T_{19}	Amb pCO_2 no NO_3^-	35.07	25.60	7.91	6.58	-	-	-	0.16
	High pCO_2 no NO_3	34.97	25.63	7.76	6.70	-	-	-	0.97
	High pCO_2 High NO_3	34.90	25.53	7.71	6.56	-	-	-	8.43
	Amb pCO_2 High NO_3	34.97	25.50	8.04	6.66	-	-	-	7.69
	High pCO_2 Mod NO_3	34.90	25.47	7.76	6.64	-	-	-	3.72
	Amb pCO_2 Mod NO_3	35.03	25.60	8.04	6.69	-	-	-	3.90
T_{20}	Amb pCO_2 no NO_3^-	34.90	25.63	7.89	6.91	1436.92	0.33	0.53	-
	High pCO_2 no NO_3	34.87	25.60	7.78	6.99	1879.81	0.23	0.96	-
	High pCO_2 High NO_3	34.80	25.53	7.74	6.93	1842.16	0.24	0.21	-

	Amb pCO_2 High NO_3	34.80	25.50	7.98	6.94	1536.59	0.25	0.19	-
	High pCO_2 Mod NO_3	34.83	25.50	7.75	6.87	1906.54	0.21	0.02	-
	Amb pCO_2 Mod NO_3	34.80	25.63	7.99	7.00	1636.82	0.30	0.10	-
T_{23}	Amb pCO_2 no NO_3^-	35.10	25.60	8.07	7.04	-	-	-	-
	High pCO_2 no NO_3	35.13	25.60	7.89	6.94	-	-	-	-
	High pCO_2 High NO_3	35.03	25.57	7.85	7.01	-	-	-	-
	Amb pCO_2 High NO_3	35.03	25.53	8.18	6.95	-	-	-	-
	High pCO_2 Mod NO_3	35.07	25.50	7.86	6.91	-	-	-	-
	Amb pCO_2 Mod NO_3	35.13	25.57	8.13	6.96	-	-	-	-
T_{26}	Amb pCO_2 no NO_3^-	34.83	25.60	7.97	6.58	1708.37	0.0	2.09	-
	High pCO_2 no NO_3	34.90	25.60	7.80	6.65	2014.48	0.0	0.54	-
	High pCO_2 High NO_3	34.83	25.53	7.75	6.58	1933.87	0.0	0.36	-
	Amb pCO_2 High NO_3	34.87	25.53	8.11	6.59	1817.33	0.0	0.77	-
	High pCO_2 Mod NO_3	34.90	25.47	7.78	6.57	2015.77	0.0	0.09	-
	Amb pCO_2 Mod NO_3	34.90	25.60	8.02	6.64	1799.39	0.0	0.41	-
T_{28}	Amb pCO_2 no NO_3^-	35.03	25.60	7.93	6.54	-	-	-	0.0
	High pCO_2 no NO_3	35.10	25.57	7.80	6.68	-	-	-	0.0
	High pCO_2 High NO_3	35.00	25.53	7.76	6.63	-	-	-	7.64
	Amb pCO_2 High NO_3	35.03	25.47	8.10	6.59	-	-	-	8.93
	High pCO_2 Mod NO_3	35.10	25.47	7.82	6.59	-	-	-	4.34
	Amb pCO_2 Mod NO_3	35.10	25.60	8.02	6.60	-	-	-	4.44

Supplementary Table S2. Generalized Linear Mixed Models (GLMM) results of the hard coral *Stylophora pistillata*. GLMM results for the physiological parameters of *S. pistillata* among abiotic treatments (acidification and nitrate enrichment treatments: ambient pCO_2 + no NO_3^-, high pCO_2 + no NO_3^-, ambient pCO_2 + moderate NO_3^-, high pCO_2 + moderate NO_3^-, ambient pCO_2 + high NO_3^-, high pCO_2 + high NO_3^-) and competition treatments (contact and no-contact corals or sections of corals). **a** Photosynthetic efficiency (PE) at initial (T_0) and final times (T_{28}); **b** PE at T_{28}; **c** Changes in PE (difference between contact and no-contact sections) at T_{28}; **d** Calcification rate; **e** Symbiodiniaceae density; **f** Chlorophyll-*a* concentration. ** $p < 0.001$, * $p < 0.05$

	Estimate	Std. Error	z-value	p-value
**a.** Photosynthetic efficiency				
Intercept	0.650	0.511	12.711	**<0.001****
High pCO_2 no NO_3^-	-0.048	0.721	-0.665	0.506
High pCO_2 High NO_3^-	-0.009	0.072	-0.118	0.906
Amb pCO_2 High NO_3^-	-0.036	0.072	-0.500	0.617
High pCO_2 Mod NO_3^-	-0.200	0.072	-2.797	**0.005***
Amb pCO_2 Mod NO_3^-	-0.030	0.072	-0.419	0.675
No contact	-0.007	0.062	-0.111	0.912
TF	-0.466	0.057	-8.119	**<0.001****
High pCO_2 no NO_3^-:No contact	-0.047	0.087	-0.544	0.586
High pCO_2 High NO_3^-:No contact	-0.071	0.087	-0.811	0.418
Amb pCO_2 High NO_3^-:No contact	0.008	0.087	0.095	0.924
High pCO_2 Mod NO_3^-:No contact	0.028	0.087	0.322	0.748
Amb pCO_2 Mod NO_3^-:No contact	-0.050	0.087	-0.576	0.565
High pCO_2 no NO_3^-:TF	0.090	0.081	1.110	0.267
High pCO_2 High NO_3^-:TF	-0.080	0.081	-0.986	0.324
Amb pCO_2 High NO_3^-:TF	0.108	0.081	1.327	0.184
High pCO_2 Mod NO_3^-:TF	0.249	0.081	3.090	**0.002***
Amb pCO_2 Mod NO_3^-:TF	0.047	0.081	0.585	0.558
No contact:TF	0.544	0.082	6.601	**<0.001****
High pCO_2 no NO_3^-:No contact:TF	0.002	0.117	0.014	0.989
High pCO_2 High NO_3^-:No contact:TF	0.230	0.117	1.969	0.049
Amb pCO_2 High NO_3^-:No contact:TF	-0.005	0.117	-0.045	0.964
High pCO_2 Mod NO_3^-:No contact:TF	-0.035	0.116	-0.229	0.765
Amb pCO_2 Mod NO_3^-:No contact:TF	0.076	0.116	0.654	0.513
**b.** Photosynthetic efficiency (T_{28})				
Intercept	0.183	0.053	3.443	**<0.001****
High pCO_2 no NO_3^-	0.042	0.075	0.560	0.575
High pCO_2 High NO_3^-	-0.088	0.075	-1.174	0.240
Amb pCO_2 High NO_3^-	0.072	0.075	0.949	0.342
High pCO_2 Mod NO_3^-	0.049	0.075	0.654	0.513
Amb pCO_2 Mod NO_3^-	0.012	0.075	0.228	0.819
No contact	0.537	0.067	8.036	**<0.001****
High pCO_2 no NO_3^-:No contact	-0.015	0.095	-0.160	0.873
High pCO_2 High NO_3^-:No contact	0.104	0.094	1.104	0.270
Amb pCO_2 High NO_3^-:No contact	-0.001	0.095	-0.005	0.996
High pCO_2 Mod NO_3^-:No contact	-0.034	0.095	-0.359	0.720
Amb pCO_2 Mod NO_3^-:No contact	0.051	0.095	0.542	0.588
**c.** Photosynthetic efficiency (difference)				
Intercept	0.127	0.017	7.674	**<0.001****
High pCO_2 no NO_3^-	-0.004	0.023	-0.166	0.868
High pCO_2 High NO_3^-	0.025	0.023	1.083	0.279

Amb pCO_2 High NO_3^-	-0.002	0.023	-0.095	0.925
High pCO_2 Mod NO_3^-	-0.008	0.023	-0.355	0.723
Amb pCO_2 Mod NO_3^-	-0.011	0.023	0.449	0.653

d. Calcification rate

Intercept	-3.229	0.143	-22.557	**<0.001****
High pCO_2 no NO_3^-	-0.590	0.202	-2.915	**0.004***
High pCO_2 High NO_3^-	-0.182	0.202	-0.899	0.365
Amb pCO_2 High NO_3^-	0.093	0.202	0.461	0.647
High pCO_2 Mod NO_3^-	-0.429	0.202	-2.119	**0.034***
Amb pCO_2 Mod NO_3^-	-0.053	0.202	-0.260	0.795
No contact	0.055	0.202	-0.270	0.788
High pCO_2 no NO_3^-:No contact	0.376	0.286	1.314	0.190
High pCO_2 High NO_3^-:No contact	-0.050	0.286	-0.175	0.861
Amb pCO_2 High NO_3^-:No contact	-0.044	0.286	-0.154	0.878
High pCO_2 Mod NO_3^-:No contact	0.118	0.286	0.411	0.681
Amb pCO_2 Mod NO_3^-:No contact	0.196	0.286	0.683	0.495

e. Symbiodiniaceae density

Intercept	1.167	0.170	6.878	**<0.001****
High pCO_2 no NO_3^-	0.360	0.217	1.655	0.098
High pCO_2 High NO_3^-	0.413	0.211	1.955	0.051
Amb pCO_2 High NO_3^-	-0.020	0.236	-0.084	0.933
High pCO_2 Mod NO_3^-	-0.014	0.232	-0.062	0.951
Amb pCO_2 Mod NO_3^-	0.210	0.220	0.954	0.340
No contact	1.564	0.183	8.570	**<0.001****
High pCO_2 no NO_3^-:No contact	-0.517	0.241	-2.141	**0.032***
High pCO_2 High NO_3^-:No contact	-0.305	0.231	-1.318	0.187
Amb pCO_2 High NO_3^-:No contact	-0.229	0.261	-0.876	0.381
High pCO_2 Mod NO_3^-:No contact	-0.176	0.256	-0.687	0.492
Amb pCO_2 Mod NO_3^-:No contact	-0.254	0.241	-1.052	0.293

f. Chlorophyll-*a* concentration

Intercept	-2.227	0.334	-6.663	**<0.001****
High pCO_2 no NO_3^-	1.163	0.393	2.958	**0.003***
High pCO_2 High NO_3^-	1.136	0.389	2.922	**0.003***
Amb pCO_2 High NO_3^-	-0.765	0.510	-1.500	0.134
High pCO_2 Mod NO_3^-	-0.807	0.512	-1.576	0.115
Amb pCO_2 Mod NO_3^-	0.969	0.396	2.449	**0.014***
No contact	1.925	0.317	6.071	**<0.001****
High pCO_2 no NO_3^-:No contact	-1.230	0.357	-3.447	**<0.001****
High pCO_2 High NO_3^-:No contact	-0.826	0.348	-2.371	**0.018***
Amb pCO_2 High NO_3^-:No contact	0.471	0.502	0.938	0.348
High pCO_2 Mod NO_3^-:No contact	0.555	0.490	1.132	0.258
Amb pCO_2 Mod NO_3^-:No contact	-0.862	0.357	-2.417	**0.016***

Supplementary Table S3. Tukey tests results of the hard coral *Stylophora pistillata*. Tukey tests results for the physiological parameters of *S. pistillata* among abiotic treatments (acidification and nitrate enrichment treatments: ambient pCO_2 + no NO_3^-, high pCO_2 + no NO_3^-, ambient pCO_2 + moderate NO_3^-, high pCO_2 + moderate NO_3^-, ambient pCO_2 + high NO_3^-, high pCO_2 + high NO_3^-) and competition treatments (contact and no-contact corals or sections of corals). **a** Photosynthetic efficiency (PE) at the final time (T_{28}); **b** Calcification rate; **c** Symbiodiniaceae density; **d** Chlorophyll-*a* concentration. The listed pairs are significantly different. ** $p < 0.001$, * $p < 0.05$

	Estimate	Std. Error	z-value	p-value
**a.** Photosynthetic efficiency				
Amb pCO_2 no NO_3^- x No contact - Amb pCO_2 no NO_3^- x Contact	0.537	0.067	8.036	**<0.001****
High pCO_2 no NO_3^- x No contact - Amb pCO_2 no NO_3^- x Contact	0.564	0.077	7.306	**<0.001****
High pCO_2 High NO_3^- x No contact - Amb pCO_2 no NO_3^- x Contact	0.553	0.077	7.167	**<0.001****
Amb pCO_2 High NO_3^- x No contact - Amb pCO_2 no NO_3^- x Contact	0.608	0.077	7.851	**<0.001****
High pCO_2 Mod NO_3^- x No contact - Amb pCO_2 no NO_3^- x Contact	0.552	0.077	7.159	**<0.001****
Amb pCO_2 Mod NO_3^- x No contact - Amb pCO_2 no NO_3^- x Contact	0.605	0.077	7.821	**<0.001****
Amb pCO_2 no NO_3^- x No contact - High pCO_2 no NO_3^- x Contact	0.495	0.077	6.415	**<0.001****
High pCO_2 no NO_3^- x No contact - High pCO_2 no NO_3^- x Contact	0.522	0.067	7.782	**<0.001****
High pCO_2 High NO_3^- x No contact - High pCO_2 no NO_3^- x Contact	0.510	0.077	6.615	**<0.001****
Amb pCO_2 High NO_3^- x No contact - High pCO_2 no NO_3^- x Contact	0.566	0.077	7.300	**<0.001****
High pCO_2 Mod NO_3^- x No contact - High pCO_2 no NO_3^- x Contact	0.510	0.077	6.607	**<0.001****
Amb pCO_2 Mod NO_3^- x No contact - High pCO_2 no NO_3^- x Contact	0.563	0.077	7.270	**<0.001****
Amb pCO_2 no NO_3^- x No contact - High pCO_2 High NO_3^- x Contact	0.625	0.077	8.126	**<0.001****
High pCO_2 no NO_3^- x No contact - High pCO_2 High NO_3^- x Contact	0.652	0.077	8.462	**<0.001****
High pCO_2 High NO_3^- x No contact - High pCO_2 High NO_3^- x Contact	0.641	0.067	9.601	**<0.001****
Amb pCO_2 High NO_3^- x No contact - High pCO_2 High NO_3^- x Contact	0.696	0.077	9.004	**<0.001****
High pCO_2 Mod NO_3^- x No contact - High pCO_2 High NO_3^- x Contact	0.640	0.077	8.316	**<0.001****
Amb pCO_2 Mod NO_3^- x No contact - High pCO_2 High NO_3^- x Contact	0.694	0.077	8.974	**<0.001****
Amb pCO_2 no NO_3^- x No contact - Amb pCO_2 High NO_3^- x Contact	0.465	0.077	6.032	**<0.001****
High pCO_2 no NO_3^- x No contact - Amb pCO_2 High NO_3^- x Contact	0.492	0.077	6.371	**<0.001****
High pCO_2 High NO_3^- x No contact - Amb pCO_2 High NO_3^- x Contact	0.481	0.077	6.232	**<0.001****
Amb pCO_2 High NO_3^- x No contact - Amb pCO_2 High NO_3^- x Contact	0.536	0.067	7.963	**<0.001****
High pCO_2 Mod NO_3^- x No contact - Amb pCO_2 High NO_3^- x Contact	0.481	0.077	6.224	**<0.001****
Amb pCO_2 Mod NO_3^- x No contact - Amb pCO_2 High NO_3^- x Contact	0.534	0.077	6.888	**<0.001****
Amb pCO_2 no NO_3^- x No contact - High pCO_2 Mod NO_3^- x Contact	0.487	0.077	6.323	**<0.001****
High pCO_2 no NO_3^- x No contact - High pCO_2 Mod NO_3^- x Contact	0.515	0.077	6.662	**<0.001****
High pCO_2 High NO_3^- x No contact - High pCO_2 Mod NO_3^- x Contact	0.503	0.077	6.523	**<0.001****
Amb pCO_2 High NO_3^- x No contact - High pCO_2 Mod NO_3^- x Contact	0.559	0.077	7.208	**<0.001****
High pCO_2 Mod NO_3^- x No contact - High pCO_2 Mod NO_3^- x Contact	0.503	0.067	7.508	**<0.001****
Amb pCO_2 Mod NO_3^- x No contact - High pCO_2 Mod NO_3^- x Contact	0.556	0.077	7.178	**<0.001****
Amb pCO_2 no NO_3^- x No contact - Amb pCO_2 Mod NO_3^- x Contact	0.520	0.077	6.743	**<0.001****
High pCO_2 no NO_3^- x No contact - Amb pCO_2 Mod NO_3^- x Contact	0.547	0.077	7.081	**<0.001****
High pCO_2 High NO_3^- x No contact - Amb pCO_2 Mod NO_3^- x Contact	0.536	0.077	6.942	**<0.001****
Amb pCO_2 High NO_3^- x No contact - Amb pCO_2 Mod NO_3^- x Contact	0.591	0.077	7.626	**<0.001****
High pCO_2 Mod NO_3^- x No contact - Amb pCO_2 Mod NO_3^- x Contact	0.535	0.077	6.934	**<0.001****
**b.** Calcification rate				
Amb pCO_2 High NO_3^- x Contact - High pCO_2 no NO_3^- x Contact	0.682	0.202	3.369	**0.037***
Amb pCO_2 Mod NO_3^- x No contact - High pCO_2 no NO_3^- x Contact	0.786	0.202	3.887	**<0.01***
Amb pCO_2 High NO_3^- x No contact - High pCO_2 no NO_3^- x Contact	0.692	0.202	3.420	**0.031***
**c.** Symbiodiniaceae density				
Amb pCO_2 no NO_3^- x No contact - Amb pCO_2 no NO_3^- x Contact	1.564	0.183	8.570	**<0.001****

High pCO_2 no NO_3^- x No contact - Amb pCO_2 no NO_3^- x Contact	1.407	0.182	7.727	**<0.001****
High pCO_2 High NO_3^- x No contact - Amb pCO_2 no NO_3^- x Contact	1.672	0.178	9.415	**<0.001****
Amb pCO_2 High NO_3^- x No contact - Amb pCO_2 no NO_3^- x Contact	1.316	0.186	7.086	**<0.001****
High pCO_2 Mod NO_3^- x No contact - Amb pCO_2 no NO_3^- x Contact	1.374	0.184	7.467	**<0.001****
Amb pCO_2 Mod NO_3^- x No contact - Amb pCO_2 no NO_3^- x Contact	1.520	0.180	8.440	**<0.001****
Amb pCO_2 no NO_3^- x No contact - High pCO_2 no NO_3^- x Contact	1.205	0.166	7.269	**<0.001****
High pCO_2 no NO_3^- x No contact - High pCO_2 no NO_3^- x Contact	1.048	0.165	6.337	**<0.001****
High pCO_2 High NO_3^- x No contact - High pCO_2 no NO_3^- x Contact	1.312	0.160	8.189	**<0.001****
Amb pCO_2 High NO_3^- x No contact - High pCO_2 no NO_3^- x Contact	0.956	0.169	5.647	**<0.001****
High pCO_2 Mod NO_3^- x No contact - High pCO_2 no NO_3^- x Contact	1.014	0.167	6.060	**<0.001****
Amb pCO_2 Mod NO_3^- x No contact - High pCO_2 no NO_3^- x Contact	1.160	0.163	7.116	**<0.001****
Amb pCO_2 no NO_3^- x No contact - High pCO_2 High NO_3^- x Contact	1.152	0.156	7.392	**<0.001****
High pCO_2 no NO_3^- x No contact - High pCO_2 High NO_3^- x Contact	0.995	0.156	6.395	**<0.001****
High pCO_2 High NO_3^- x No contact - High pCO_2 High NO_3^- x Contact	1.260	0.150	8.403	**<0.001****
Amb pCO_2 High NO_3^- x No contact - High pCO_2 High NO_3^- x Contact	0.903	0.160	5.646	**<0.001****
High pCO_2 Mod NO_3^- x No contact - High pCO_2 High NO_3^- x Contact	0.961	0.158	6.096	**<0.001****
Amb pCO_2 Mod NO_3^- x No contact - High pCO_2 High NO_3^- x Contact	1.107	0.153	7.237	**<0.001****
Amb pCO_2 no NO_3^- x No contact - Amb pCO_2 High NO_3^- x Contact	1.584	0.192	8.262	**<0.001****
High pCO_2 no NO_3^- x No contact - Amb pCO_2 High NO_3^- x Contact	1.427	0.191	7.464	**<0.001****
High pCO_2 High NO_3^- x No contact - Amb pCO_2 High NO_3^- x Contact	1.692	0.187	9.041	**<0.001****
Amb pCO_2 High NO_3^- x No contact - Amb pCO_2 High NO_3^- x Contact	1.336	0.194	6.871	**<0.001****
High pCO_2 Mod NO_3^- x No contact - Amb pCO_2 High NO_3^- x Contact	1.394	0.193	7.222	**<0.001****
Amb pCO_2 Mod NO_3^- x No contact - Amb pCO_2 High NO_3^- x Contact	1.539	0.189	8.131	**<0.001****
Amb pCO_2 no NO_3^- x No contact - High pCO_2 Mod NO_3^- x Contact	1.579	0.187	8.464	**<0.001****
High pCO_2 no NO_3^- x No contact - High pCO_2 Mod NO_3^- x Contact	1.422	0.186	7.641	**<0.001****
High pCO_2 High NO_3^- x No contact - High pCO_2 Mod NO_3^- x Contact	1.686	0.182	9.280	**<0.001****
Amb pCO_2 High NO_3^- x No contact - High pCO_2 Mod NO_3^- x Contact	1.330	0.189	7.021	**<0.001****
High pCO_2 Mod NO_3^- x No contact - High pCO_2 Mod NO_3^- x Contact	1.388	0.188	7.389	**<0.001****
Amb pCO_2 Mod NO_3^- x No contact - High pCO_2 Mod NO_3^- x Contact	1.534	0.184	8.334	**<0.001****
Amb pCO_2 no NO_3^- x No contact - Amb pCO_2 Mod NO_3^- x Contact	1.355	0.168	8.084	**<0.001****
High pCO_2 no NO_3^- x No contact - Amb pCO_2 Mod NO_3^- x Contact	1.198	0.167	7.161	**<0.001****
High pCO_2 High NO_3^- x No contact - Amb pCO_2 Mod NO_3^- x Contact	1.463	0.162	9.020	**<0.001****
Amb pCO_2 High NO_3^- x No contact - Amb pCO_2 Mod NO_3^- x Contact	1.107	0.171	6.459	**<0.001****
High pCO_2 Mod NO_3^- x No contact - Amb pCO_2 Mod NO_3^- x Contact	1.165	0.169	6.879	**<0.001****
Amb pCO_2 Mod NO_3^- x No contact - Amb pCO_2 Mod NO_3^- x Contact	1.311	0.165	7.943	**<0.001****
High pCO_2 High NO_3^- x No contact - Amb pCO_2 High NO_3^- x No contact	0.356	0.104	3.420	**0.027***

d. Chlorophyll-*a* concentration

Amb pCO_2 no NO_3^- x No contact - Amb pCO_2 no NO_3^- x Contact	1.925	0.317	6.071	**<0.001****
High pCO_2 no NO_3^- x No contact - Amb pCO_2 no NO_3^- x Contact	1.858	0.378	4.921	**<0.001***
Amb pCO_2 Mod NO_3^- x No contact - Amb pCO_2 no NO_3^- x Contact	2.031	0.376	5.409	**<0.001****
High pCO_2 Mod NO_3^- x No contact - Amb pCO_2 no NO_3^- x Contact	1.673	0.383	4.366	**<0.001***
Amb pCO_2 High NO_3^- x No contact - Amb pCO_2 no NO_3^- x Contact	1.631	0.391	4.171	**0.002***
High pCO_2 High NO_3^- x No contact - Amb pCO_2 no NO_3^- x Contact	2.236	0.374	5.981	**<0.001****
High pCO_2 Mod NO_3^- x Contact - High pCO_2 no NO_3^- x Contact	-1.970	0.450	-4.382	**<0.001****
Amb pCO_2 High NO_3^- x Contact - High pCO_2 no NO_3^- x Contact	-1.928	0.449	-4.290	**<0.001****
High pCO_2 no NO_3^- x No contact - High pCO_2 no NO_3^- x Contact	0.695	0.165	4.217	**0.001***
High pCO_2 High NO_3^- x No contact - High pCO_2 no NO_3^- x Contact	1.073	0.271	3.963	**0.004***
High pCO_2 Mod NO_3^- x Contact - Amb pCO_2 Mod NO_3^- x Contact	-1.776	0.452	-3.932	**0.004***
Amb pCO_2 no NO_3^- x No contact - Amb pCO_2 Mod NO_3^- x Contact	0.956	0.278	3.443	**0.023***
Amb pCO_2 Mod NO_3^- x No contact - Amb pCO_2 Mod NO_3^- x Contact	1.063	0.166	6.404	**<0.001****
High pCO_2 High NO_3^- x No contact - Amb pCO_2 Mod NO_3^- x Contact	1.267	0.275	4.615	**<0.001****
High pCO_2 High NO_3^- x Contact - High pCO_2 Mod NO_3^- x Contact	1.943	0.446	4.357	**<0.001****
Amb pCO_2 no NO_3^- x No contact - High pCO_2 Mod NO_3^- x Contact	2.732	0.439	6.227	**<0.001****
High pCO_2 no NO_3^- x No contact - High pCO_2 Mod NO_3^- x Contact	2.665	0.437	6.094	**<0.001****
Amb pCO_2 Mod NO_3^- x No contact - High pCO_2 Mod NO_3^- x Contact	2.838	0.436	6.513	**<0.001****
High pCO_2 Mod NO_3^- x No contact - High pCO_2 Mod NO_3^- x Contact	2.480	0.390	6.356	**<0.001****

Amb pCO_2 High NO_3^- x No contact - High pCO_2 Mod NO_3^- x Contact	2.438	0.448	5.443	**<0.001****
High pCO_2 High NO_3^- x No contact - High pCO_2 Mod NO_3^- x Contact	3.043	0.434	7.003	**<0.001****
High pCO_2 High NO_3^- x Contact - Amb pCO_2 High NO_3^- x Contact	1.901	0.446	4.264	**<0.001****
Amb pCO_2 no NO_3^- x No contact - Amb pCO_2 High NO_3^- x Contact	2.689	0.440	6.112	**<0.001****
High pCO_2 no NO_3^- x No contact - Amb pCO_2 High NO_3^- x Contact	2.622	0.438	5.987	**<0.001****
Amb pCO_2 Mod NO_3^- x No contact - Amb pCO_2 High NO_3^- x Contact	2.796	0.437	6.403	**<0.001****
High pCO_2 Mod NO_3^- x No contact - Amb pCO_2 High NO_3^- x Contact	2.437	0.442	5.508	**<0.001****
Amb pCO_2 High NO_3^- x No contact - Amb pCO_2 High NO_3^- x Contact	2.395	0.411	5.832	**<0.001****
High pCO_2 High NO_3^- x No contact - Amb pCO_2 High NO_3^- x Contact	3.000	0.435	6.890	**<0.001****
Amb pCO_2 Mod NO_3^- x No contact - High pCO_2 High NO_3^- x Contact	0.895	0.267	3.348	**0.033***
High pCO_2 High NO_3^- x No contact - High pCO_2 High NO_3^- x Contact	1.099	0.148	7.450	**<0.001****

Supplementary Table S4. Generalized Linear Mixed Models (GLMM) results of the soft coral *Xenia* spp. GLMM results for the physiological parameters of *Xenia* spp. among abiotic treatments (acidification and nitrate enrichment treatments: ambient pCO_2 + no NO_3^-, high pCO_2 + no NO_3^-, ambient pCO_2 + moderate NO_3^-, high pCO_2 + moderate NO_3^-, ambient pCO_2 + high NO_3^-, high pCO_2 + high NO_3^-) and competition treatments (contact and no-contact corals). **a** Growth rate; **b** Symbiodiniaceae density; **c** Chlorophyll-*a* concentration. ** $p < 0.001$, * $p < 0.05$

	Estimate	Std. Error	z-value	p-value
**a.** Growth rate				
Intercept	-0.141	0.325	-4.330	**<0.001*** **
High pCO_2 no NO_3^-	0.011	0.046	0.234	0.815
High pCO_2 High NO_3^-	0.030	0.046	0.662	0.508
Amb pCO_2 High NO_3^-	-0.028	0.046	-0.609	0.543
High pCO_2 Mod NO_3^-	0.039	0.046	0.858	0.391
Amb pCO_2 Mod NO_3^-	-0.030	0.046	-0.660	0.509
No contact	0.063	0.028	2.235	**0.025***
High pCO_2 no NO_3:No contact	0.022	0.040	0.554	0.580
High pCO_2 High NO_3^-:No contact	0.013	0.040	0.333	0.739
Amb pCO_2 High NO_3^-:No contact	0.102	0.040	2.560	**0.011***
High pCO_2 Mod NO_3^-:No contact	-0.055	0.040	-1.388	0.165
Amb pCO_2 Mod NO_3^-:No contact	-0.013	0.040	-0.333	0.739
**b.** Symbiodiniaceae density				
Intercept	-0.098	0.286	-0.342	0.733
High pCO_2 no NO_3^-	-0.012	0.398	-0.028	0.978
High pCO_2 High NO_3^-	0.702	0.405	1.732	0.083
Amb pCO_2 High NO_3^-	0.555	0.397	1.398	0.162
High pCO_2 Mod NO_3^-	0.227	0.406	0.560	0.575
Amb pCO_2 Mod NO_3^-	0.090	0.397	0.227	0.821
No contact	0.104	0.284	0.368	0.713
High pCO_2 no NO_3:No contact	-0.139	0.404	-0.344	0.731
High pCO_2 High NO_3^-:No contact	0.016	0.405	0.040	0.969
Amb pCO_2 High NO_3^-:No contact	-0.968	0.397	-2.440	**0.015 ***
High pCO_2 Mod NO_3^-:No contact	-0.231	0.406	-0.567	0.571
Amb pCO_2 Mod NO_3^-:No contact	-0.440	0.394	-1.118	0.264
**c.** Chlorophyll-*a* concentration				
Intercept	-0.466	0.621	-0.751	0.453
High pCO_2 no NO_3^-	0.826	0.540	1.529	0.126
High pCO_2 High NO_3^-	-0.120	0.602	-0.199	0.842
Amb pCO_2 High NO_3^-	1.586	0.508	3.120	**0.002***
High pCO_2 Mod NO_3^-	0.763	0.559	1.365	0.172
Amb pCO_2 Mod NO_3^-	0.978	0.550	1.779	0.075
No contact	1.479	0.463	3.197	**0.001***
High pCO_2 no NO_3:No contact	-0.999	0.504	-1.981	0.048
High pCO_2 High NO_3^-:No contact	0.220	0.581	0.378	0.705
Amb pCO_2 High NO_3^-:No contact	-1.351	0.447	-3.021	**0.003***
High pCO_2 Mod NO_3^-:No contact	-1.037	0.545	-1.901	0.057
Amb pCO_2 Mod NO_3^-:No contact	-0.645	0.517	-1.250	0.211

Supplementary Table S5. Tukey tests results of the soft coral *Xenia* spp. Tukey tests results for the physiological parameters of *Xenia* spp. among abiotic treatments (acidification and nitrate enrichment treatments: ambient pCO_2 + no NO_3^-, high pCO_2 + no NO_3^-, ambient pCO_2 + moderate NO_3^-, high pCO_2 + moderate NO_3^-, ambient pCO_2 + high NO_3^-, high pCO_2 + high NO_3^-) and competition treatments (contact and no-contact corals). **a** Growth rate; **b** Chlorophyll-*a* concentration. The listed pairs are significantly different. ** $p < 0.01$, * $p < 0.05$

	Estimate	Std. Error	z-value	p-value
**a.** Growth rate				
Amb pCO_2 High NO_3 x No contact - Amb pCO_2 Mod NO_3^- x Contact	0.167	0.046	3.627	**0.014***
Amb pCO_2 High NO_3 x No contact - Amb pCO_2 High NO_3^- x Contact	0.164	0.028	5.855	**<0.01****
**b.** Chlorophyll-*a* concentration				
Amb pCO_2 Mod NO_3^- x No contact - Amb pCO_2 no NO_3^- x Contact	1.811	0.509	3.558	**0.018***
Amb pCO_2 Mod NO_3^- x No contact - High pCO_2 High NO_3^- x Contact	1.931	0.540	3.579	**0.016***
High pCO_2 High NO_3^- x No contact - High pCO_2 High NO_3^- x Contact	1.699	0.475	3.576	**0.016***
